# The independence of impairments in proprioception and visuomotor adaptation after stroke

**DOI:** 10.1186/s12984-024-01360-7

**Published:** 2024-05-18

**Authors:** Robert T. Moore, Mark A. Piitz, Nishita Singh, Sean P. Dukelow, Tyler Cluff

**Affiliations:** 1 Department of Clinical Neurosciences, Cumming School of Medicine, University of Calgary, 3330 Hospital Dr NW, Calgary, AB Canada; 2 Hotchkiss Brain Institute, University of Calgary, 3330 Hospital Dr NW, Calgary, AB Canada; 3 Faculty of Kinesiology, University of Calgary, 2500 University Dr NW, Calgary, AB Canada

**Keywords:** Stroke, Rehabilitation, Motor learning, Proprioception, Motor adaptation, Upper limb, Robotics

## Abstract

**Background:**

Proprioceptive impairments are common after stroke and are associated with worse motor recovery and poor rehabilitation outcomes. Motor learning may also be an important factor in motor recovery, and some evidence in healthy adults suggests that reduced proprioceptive function is associated with reductions in motor learning. It is unclear how impairments in proprioception and motor learning relate after stroke. Here we used robotics and a traditional clinical assessment to examine the link between impairments in proprioception after stroke and a type of motor learning known as visuomotor adaptation.

**Methods:**

We recruited participants with first-time unilateral stroke and controls matched for overall age and sex. Proprioceptive impairments in the more affected arm were assessed using robotic arm position- (APM) and movement-matching (AMM) tasks. We also assessed proprioceptive impairments using a clinical scale (Thumb Localization Test; TLT). Visuomotor adaptation was assessed using a task that systematically rotated hand cursor feedback during reaching movements (VMR). We quantified how much participants adapted to the disturbance and how many trials they took to adapt to the same levels as controls. Spearman’s rho was used to examine the relationship between proprioception, assessed using robotics and the TLT, and visuomotor adaptation. Data from healthy adults were used to identify participants with stroke who were impaired in proprioception and visuomotor adaptation. The independence of impairments in proprioception and adaptation were examined using Fisher’s exact tests.

**Results:**

Impairments in proprioception (58.3%) and adaptation (52.1%) were common in participants with stroke (n = 48; 2.10% acute, 70.8% subacute, 27.1% chronic stroke). Performance on the APM task, AMM task, and TLT scores correlated weakly with measures of visuomotor adaptation. Fisher’s exact tests demonstrated that impairments in proprioception, assessed using robotics and the TLT, were independent from impairments in visuomotor adaptation in our sample.

**Conclusion:**

Our results suggest impairments in proprioception may be independent from impairments in visuomotor adaptation after stroke. Further studies are needed to understand factors that influence the relationship between motor learning, proprioception and other rehabilitation outcomes throughout stroke recovery.

**Supplementary Information:**

The online version contains supplementary material available at 10.1186/s12984-024-01360-7.

## Background

Stroke is a neurological disease that can result in a myriad of impairments. Although motor impairments are most common (80% of cases) [1], the majority of individuals with stroke (50–65%) also experience proprioceptive impairments affecting the sense of body position (position sense) and/or motion (kinesthetic sense) [2–4]. Despite growing recognition that proprioceptive impairments are associated with poorer motor recovery and rehabilitation outcomes [1, 5, 6], therapy tends to focus on improving the performance of movements that are important for daily living (e.g., feeding or grooming). It is unclear how proprioceptive impairments interact with the capacity to improve arm movements with practice after stroke.

Motor learning is a broad term that encompasses a variety of neural and behavioural processes that support long-term motor skill learning and short-term changes in behaviour that result from motor adaptation [7]. Skill learning involves processes that support the acquisition of new motor skills and sequences of movements. These movements are acquired over prolonged periods of practice and require retention to maintain skilled performance. Motor adaptation describes processes that are engaged by feedback mechanisms that eliminate errors and help to maintain the performance of skilled actions in different environments, tasks, and contexts [8–15]. Some evidence indicates these adaptive mechanisms may also be supported by neuroanatomical changes that help to retain and quickly engage memories of different environments, tasks, and contexts [16].

Rehabilitation interventions are generally based on the premise that motor learning is possible after stroke. Indeed, it is widely accepted that a variety of motor learning processes could be important for motor recovery [17, 18], and many forms of therapy attempt to leverage motor learning principles to promote neuroplasticity and facilitate the recovery of motor function in clinical settings [19]. The approach may be too simplistic. Growing evidence indicates that a wide range of motor learning impairments can occur after stroke, impacting the ability to learn sequences of arm and finger movements [20], perform tracking tasks [21, 22], and adapt reaching movements to counter visual [23–27] and force disturbances that disrupt movement accuracy [28–30].

Our understanding of how proprioceptive impairments interact with the ability to adapt and improve the performance of arm movements after stroke is limited. Much of our understanding comes from studies in healthy adults that have yielded mixed results. One study in healthy older adults reported that greater variability in position matching was associated with slower adaptation to a visuomotor rotation [31]. In contrast, others have reported that greater variability in position matching was associated with greater implicit adaptation in younger adults [32]. Finally, other work did not observe significant relationships between position or kinesthetic sense and visuomotor adaptation in healthy young or older adults [33]. These results have spurred an ongoing debate over the importance of proprioceptive function in motor adaptation.

Stroke may be a useful model for understanding how motor adaptation changes when proprioception is impaired. One study in chronic stroke demonstrated that reduced position sense was associated with reduced capacity to adapt and counter forces that disturb the arm during reaching movements [29]. It is unclear if a similar relationship exists between position sense and the capacity to adapt to visual disturbances since the processes that support adaptation to visual and force disturbances may rely on distinct behavioural mechanisms [13, 34] and neural structures [35, 36]. The relationship between kinesthetic sense and motor adaptation is also a relatively unexplored but important question since kinesthesia may rely on distinct neuroanatomical pathways from position sense [4, 37–39]. Characterizing the relationship between proprioceptive impairments and motor adaptation may be an important step in understanding factors that interact with the ability to adapt and improve the performance of upper limb movements after stroke.

Here we used a robotic device to examine the relationship between proprioception, assessed using bilateral position- and movement-matching tasks, and a specific type of motor learning known as visuomotor adaptation. Visuomotor adaptation describes the process of modifying movements in response to errors caused by a visual disturbance that systematically disrupts the relationship between the participant’s arm movements and visual feedback displayed in their workspace. This type of adaptation is thought to resemble challenges that individuals with stroke encounter on a daily basis while brushing their teeth in a mirror or using a computer mouse to guide a digital cursor on a screen [26]. Our objective was to examine the relationship between proprioception, assessed using robotic tasks and a traditional clinical scale (TLT—Thumb Localization Test), and measures of visuomotor adaptation after stroke.

## Methods

### Participants

Adults with a diagnosis of stroke were recruited from the rehabilitation units at the Foothills Medical Centre and Dr. Vernon Fanning Centre in Calgary, AB, Canada. We also recruited participants who were discharged from the rehabilitation units but consented to being contacted for research. Inclusion criteria were: clinical diagnosis of first-time, unilateral ischemic or hemorrhagic stroke (confirmed by neuroimaging) and the ability to follow simple task instructions. Exclusion criteria were: history of prior stroke, cerebellar stroke, concomitant OR chronic neurological conditions (e.g., cerebellar ataxia, Parkinson’s disease, myasthenia gravis), upper-limb musculoskeletal injuries that could impede on their ability to perform the experimental tasks, difficulty understanding and/or following instructions, or the presence of motor apraxia [40]. The ability to follow task instructions was verified by consulting with clinical staff and therapists in the stroke units. Participants who were recruited after being discharged from the rehabilitation units were assessed by research staff for their ability to follow task instructions. In all cases, the ability to follow task instructions was verified by research staff during a brief familiarization period preceding each task. We recruited control participants from the community at the University of Calgary and greater Calgary area. Controls were eligible to participate if they had no history of stroke or other neurological conditions, and no recent or active upper-limb musculoskeletal injuries that would interfere with participating in the study. Participants provided written informed consent to protocols approved by the Conjoint Health Research Ethics Board at the University of Calgary prior to performing the experiment (REB15-1086).

### Robotic apparatus

**Fig. 1:**
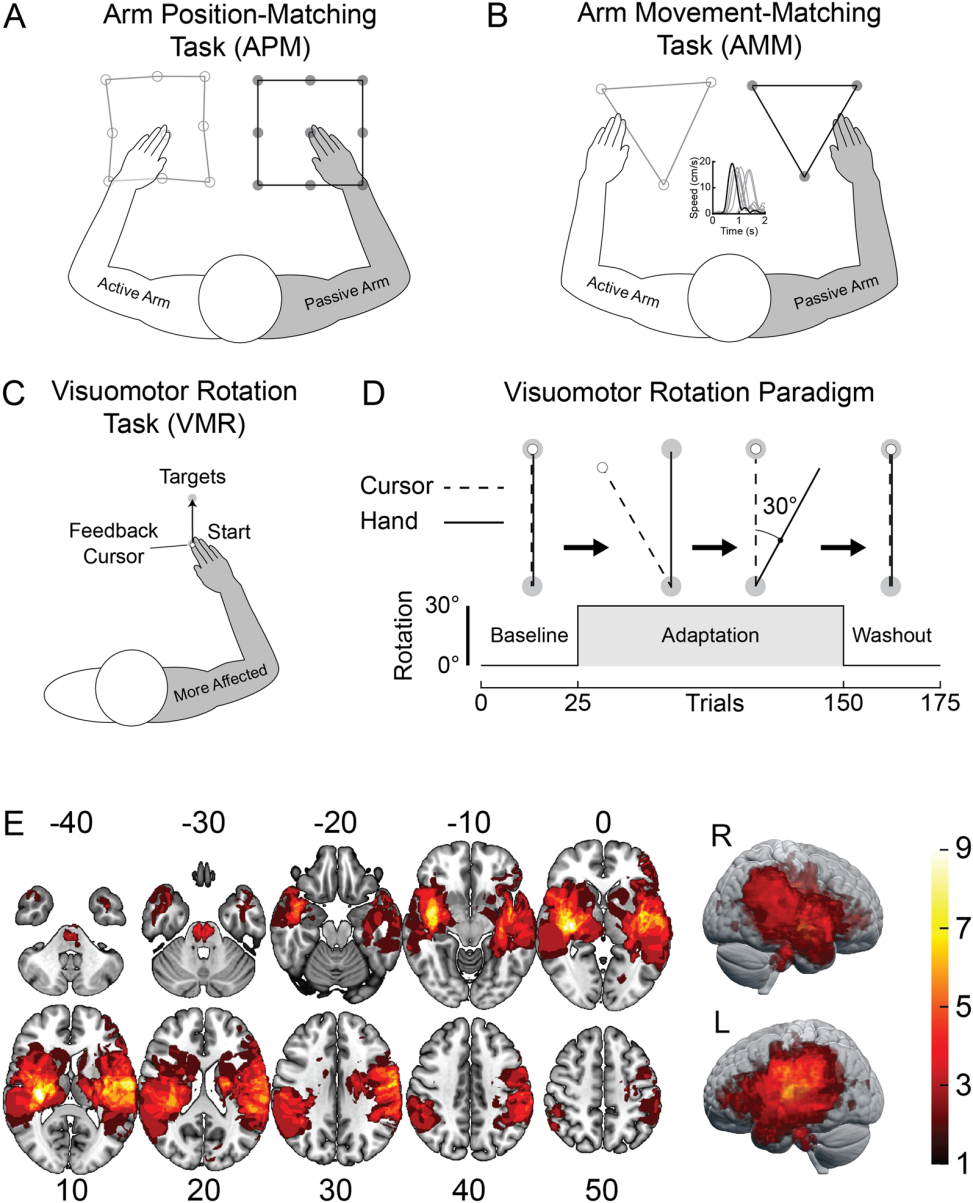
General task designs and lesion characteristics. **A** Arm position-matching task (APM). The robot moved the participant’s more-affected arm (passive arm) to one of nine positions. Participants were instructed to mirror match the position of their passive arm with their less-affected arm (active arm). **B** Arm movement-matching task (AMM). The robot moved the participant’s more-affected arm (passive arm) to one of three positions. Participants were instructed to match the speed and direction of the robot with their active arm. The APM and AMM tasks were performed in absence of direct vision of the arms and hands. **C** Participants made reaching movements from a start target located in front of their body to a singular end target 10 cm away (VMR). **D** Time course of VMR task showing baseline, adaptation, and washout phases. **E** Lesion characteristics for the participants with stroke (neurological display convention; n = 47). MNI coordinates are presented for each axial image slice. Red regions indicate regions in which fewer participants had stroke-related damage. Yellow indicates regions with more stroke-related damage

Schematics of the robotic tasks are depicted in Fig. 1. Participants were seated in a robotic exoskeleton with their arms supported against gravity by adjustable linkages (Kinarm, Kingston, ON, Canada). The apparatus allowed them to move their arms in a near-frictionless environment while interacting with targets projected on a virtual reality display. Participants were shown a 0.8 cm diameter hand-feedback cursor calibrated to the position of their index fingertip. Direct vision of the arm and hand were blocked throughout the experiment using a metal shutter. Participants also wore a cloth bib to block direct vision of the upper arms and shoulders. The experiment consisted of an arm position-matching task (APM) to assess position sense [41], an arm movement-matching task (AMM) to assess kinesthetic sense [2], and a visuomotor rotation task (VMR) to assess visuomotor adaptation [26].

### Arm position-matching task (APM)

The APM task assesses judgments of static arm position and has been validated for the assessment of position sense in adults with stroke [1, 4, 6, 41–49]. At the beginning of each trial, the robot moved the participant’s more-affected arm to one of nine positions spaced 10 cm apart (passive arm; Fig. 1A). The robot moved directly to the target and followed a bell-shaped speed profile. The average peak speed of the passive arm was calculated for each participant across targets. The group mean was 0.283 m/s [range of participant means: 0.167–0.406 m/s]) [41]. The nine positions were organized within a 3 × 3 grid in which the central position was located such that the arm was at 30° shoulder flexion and 90° elbow flexion [41]. Participants moved their opposite arm (active arm) so that their hand was in the mirror-opposite position of the passive arm. The participant verbally indicated to the robot operator when they had mirror-matched the position of the passive arm with their active arm. The operator then triggered the next trial, and the passive arm was moved to the next position in the protocol without returning to the centre of the grid. The nine positions were presented in random order within each block. Blocks were pseudorandomized such that participants would not encounter the same position in two consecutive trials. The task was comprised of six blocks for a total of 54 trials.

We quantified performance on the APM task using four variables thought to reflect distinct types of errors commonly made by individuals with impaired position sense (see KST summary, www.kinarm.com) [6, 41, 44].

1. ***Absolute Error (AE***_***xy***_***):*** Absolute error is a common method for assessing the accuracy of position sense judgments [3, 50–52]. We quantified the *Absolute Error* in the *x-* and *y-*directions between the matched position of the active arm and the position of the passive arm averaged across all trials. *AE*_*xy*_ provides a general measure of position-matching ability [44, 53, 54]. Larger *AE*_*xy*_ values reflect worse performance in the APM task [44].
2. ***Variability (Var***_***xy***_***):*** This measure quantifies trial-to-trial variability in the matched location of the active arm (*Var*_*xy*_). Greater *Var*_*xy*_ reflects worse performance and has been shown to be elevated following damage to sensory areas of the brain [46].
3. ***Area (Area***_***xy***_***):*** This measure quantifies the area covered by the matched positions of the active arm relative to the region enclosed by the passive arm moved by the robot (*Area*_*xy*_). The *Area*_*xy*_ variable characterizes impairments in spatial awareness which contribute to biases in perceiving specific regions of the workspace [41]. An *Area*_*xy*_ value less than one indicates the participant perceives a contracted representation of their workspace, while values larger than one indicate the participant perceives an expanded representation of their workspace [47].
4. ***Spatial Shift (Shift***_***xy***_***):*** Deficits in spatial awareness may create a shift in the perceived location of the target set [41]. The *Spatial Shift* parameter quantifies systematic biases in the area covered by the active arm relative to the passive arm (*Shift*_*xy*_). A *Shift*_*xy*_ value of zero indicates there was no translation of the workspace of the active arm compared to the passive arm moved by the robot. *Shift*_*xy*_ values that are smaller (leftward or downward shift) or larger (rightward or upward shift) than zero reflect greater translation of the perceived workspace (both *x*- and *y*-directions).

We also examined a global measure of performance (*APM Task Score*) based on the *x*- and *y*-directions of the APM variables listed above (Additional file 1).

5. ***APM Task Score:*** The task score provides a global measure of position sense (*APM Task Score*) using the variables derived from the APM task. The procedure is automated by the Dexterit-E software (Kinarm™, Kingston, ON). Here we provide a summary of the process (additional details available in Kinarm Standard Task (KST) summary, www.kinarm.com). Each participant’s score was calculated using existing normative datasets for the APM task that accounted for age, sex, and handedness (2221 exams, N = 797, 434 female, age = [18–93]). First, the four variables were decomposed into *x*- and *y*-directions and converted to *z*-scores based on the normative data. *Area* and *Shift* are two-sided variables, meaning a value of 1 (*Area*) or 0 (*Shift*) are ideal and smaller or larger values reflect impaired performance. *Area* and *Shift* were left as *z*-scores in the calculation of the *APM Task Score*. One-sided variables in which a high value is abnormal (i.e., *AE* and *Var*) were converted to *zeta*-scores (a one-sided transformation to *z*-score data) so that the best possible score was 0 and higher values were associated with worse performance (see Kinarm Standard Task (KST) summary, www.kinarm.com). *Z*- and *zeta*-scores were then used to calculate the Root-Sum-Square (RSS) across all task variables [49, 55]. The RSS distances were renormalized to *z*-scores using Box-Cox transformations [56]. Finally, *z*-scores were converted to *zeta*-scores (one tailed *z*-distributions) so that an *APM Task Score* greater than 1.96 was outside of 95% of the normative range [49, 55] and considered an impairment in position sense.

### Arm movement-matching task (AMM)

The AMM task assesses the ability to sense arm motion and has been validated for the assessment of kinesthetic sense in adults with stroke [2, 4]. The robot moved the participant’s more-affected arm to one of three possible positions at the beginning of each trial (passive arm; Fig. 1B). The targets were organized in a triangle with the center positioned at a configuration of 30° shoulder flexion and 90° elbow flexion. At the beginning of each trial, a red target appeared in the mirror-opposite position on the virtual display. Participants were instructed to guide a hand-feedback cursor aligned to the tip of the index finger of their opposite, less-affected arm (active arm) into the target. This ensured that trials began with the arms in approximately mirrored start positions. The visual target and cursor then disappeared from the screen. After a random delay (1500 ± 250 ms, uniformly distributed), the robot moved the passive arm (more affected by stroke) 20 cm to one of the two other target locations. The robot moved the passive arm with a bell-shaped speed profile that peaked at approximately 20 cm/s. Subjects were instructed to mirror-match the speed and direction of the robot’s movement with their active arm (less affected by stroke). One block consisted of all six possible combinations of movements. The blocks were pseudorandomized such that participants would not reach to the same target in consecutive trials. Participants performed six blocks for a total of 36 movements.

We quantified performance on the AMM task using four variables (see KST summary, www.kinarm.com) [1, 2]. We also quantified an *AMM Task Score* as a global measure of performance on the AMM task.

1. ***Response Latency (RL):*** Response latency was quantified as the time difference in movement onset for the passive and active arm (*RL*). Longer *RL* reflects worse performance [2].
2. ***Peak Speed Ratio (PSR):*** The *PSR* is defined as the ratio between the peak speeds of the passive arm (robot moved) compared to the active arm on each trial (*PSR*). A *PSR* of less than one indicates the active arm moved slower than the passive arm and values larger than one indicate the active arm moved faster than the passive arm.
3. ***Initial Direction Error (IDE):*** The *IDE* is the absolute difference in initial direction of movement (measured at peak hand speed) for the passive and active arms. An *IDE* equal to zero reflects mirror opposite movements and larger values indicate greater differences in movement directions between the arms.
4. ***Path Length Ratio (PLR):*** The *PLR* reflects the ratio of the distance of movement in the passive and active arms (*PLR*). A *PLR* value less than one indicates the active arm moved less than the passive arm, whereas values larger than one indicate the active arm moved farther than the passive arm.
5. ***AMM Task Score:*** A global measure of the kinesthetic sense (*AMM Task Score*) was computed using the 4 variables derived from the AMM task as well as the number of trials failed (see Additional file 2). Normative datasets were used to calculate the *AMM Task Score* while accounting for the age, sex, and handedness of each participant (exams: 420, N = 210, 118 female, age = [18–93]). The procedure is automated by the Dexterit-E software (Kinarm™, Kingston, ON). Here we provide a summary of the process (additional details available in Kinarm Standard Task (KST) summary, www.kinarm.com). Each variable was converted to a *z*-score based on the normative data. *SPR* and *PLR* are two-sided variables meaning a score of one reflects ideal performance and higher or lower values indicate worse performance. *SPR* and *PLR* were kept as *z*-scores for the calculation of the *AMM Task Score*. Conversely, long *RL* and larger IDE are associated with impairment (one-sided variables) and were converted to *zeta*-scores (see Kinarm Standard Task (KST) summary, www.kinarm.com). *Z*- and *zeta*-scores were used to calculate the RSS [49, 55] which was then converted into a *z*-score [56] and then a *zeta*-score. Consequently, smaller scores indicate better performance and larger scores reflect poorer performance [49, 55]. An *AMM Task Score* greater than 1.96 was outside 95% of the normative data and considered an impairment in the kinesthetic sense.

### Visuomotor rotation task (VMR)

The VMR task assesses how individuals adapt their reaching movements to visual error-feedback [26]. Direct vision of the arms was blocked throughout the VMR task and participants were given information about hand position using a feedback cursor presented in their workspace. Participants began the VMR task by guiding the feedback cursor into a 2 cm diameter start target (Fig. 1C). After a random delay (750 ± 500 ms; uniformly distributed) a single end target appeared 10 cm directly in front of the start target. We instructed participants to make smooth and accurate reaching movements to the end target. Upon reaching the end target (2 cm diameter), participants were required to stabilize in this position for 1000 ms to complete the trial. The start target then reappeared and participants moved back to begin the next trial. We did not constrain reaction times and participants reached at a self-selected pace. Participants with stroke performed the task with their more affected arm as this is the arm that typically undergoes rehabilitation [57]. Controls performed the task with their dominant arm as previous research has shown similar adaptation across the arms in healthy adults [58, 59].

The overall paradigm for the VMR task is presented in Fig. 1D. We characterized the nominal reaching patterns of each participant over the course of 25 baseline trials in which participants received veridical feedback with the motion of the cursor aligned to their fingertip. Next, we abruptly rotated the relationship between the position of the feedback cursor and the participant’s fingertip by 30° counter-clockwise (adaptation phase). Consequently, forward movement of the hand resulted in the cursor traveling 30° to the left of the end target. During the adaptation phase, participants made 125 reaching movements with the rotated hand cursor-feedback. The feedback cursor was then unexpectedly realigned to the participant’s index fingertip in the washout phase. They made 25 movements with veridical feedback to washout the effects of adaptation. The length of the task was selected to avoid fatigue and was well tolerated by both controls and participants with stroke.

Visuomotor adaptation was quantified as the signed direction of hand motion, relative to a straight line between the start and end targets, measured at 150 ms after the onset of each movement. Movement onset was the time at which the participant’s forward hand velocity exceeded 12.5% of the peak hand velocity [13]. Measuring the initial reach direction of the hand 150 ms after the onset of movement allowed us to measure changes in the planned reach trajectory while limiting the influence of corrective movements that take place throughout the movement [60].

We examined performance on the *VMR* task by quantifying the average reach direction in *Initial* and *Final Adaptation* [26]. We also calculated the number of trials taken for participants to adapt to the rotation (*Trials to Adapt*).

1. ***Initial adaptation:*** *Initial Adaptation* was defined as the mean initial reach direction of the first 15 trials of the adaptation phase. This measure quantifies how much individuals adapted their movements when they first encountered the visuomotor rotation.
2. ***Final adaptation:*** *Final Adaptation* was defined as the mean initial reach direction of the last 15 trials of the adaptation phase. This measure quantifies how much individuals adapted their movements after encountering the rotation for 110 practice trials.
3. ***Trials to adapt:*** This measure reflects the number of trials that each participant required to adapt to the rotation. We first determined the 95% range of the control data for *Final Adaptation*. We used the lower bound of this range as a threshold and defined *Trials to Adapt* as the first trial to exceed this threshold for 15 consecutive trials. Failure to adapt within the normal range by the end of the adaptation phase resulted in a score of 125 (number of trials in the adaptation phase).

The VMR task is not a standard Kinarm task like the APM and AMM tasks and does not have an existing normative dataset. We started to construct a normative dataset for this task that was matched to the overall age and sex of the stroke sample. We quantified normative ranges (containing 95% of the control data) for each measure of adaptation. Individuals with *Initial* and *Final Adaptation* below 95% of the control data were considered to have impairments on these measures. Individuals requiring more *Trials to Adapt* than 95% of controls were considered impaired on this measure. Participants were considered to have impaired adaptation if they were flagged as being impaired in at least one measure of adaptation.

### Imaging and lesion delineation

Clinical MRI was obtained for 41 participants at a median of one day [range = 0–36] post-stroke. MRI was collected on a 1.5 T Siemens or 3.0 T GE Medical Systems scanner. T2 Fluid-Attenuated Inversion Recovery (FLAIR), Diffusion Weighted Imaging (DWI), and Apparent Diffusion Coefficient (ADC) sequences were acquired for all participants who underwent an MRI scan. Participants with hemorrhagic stroke also received Susceptibility Weighted Imaging (SWI) or Gradient Echo (GRE) sequences. We obtained non-contrast CT scans for a smaller proportion of participants (n = 6, median = 1, [range = 0–9]). Acute stroke imaging protocols at the Foothills Medical Centre specify that MRI is not performed when there is a clearly defined lesion in the clinical CT [37, 46]. CT scans were collected on a Siemens CT system or one of three GE CT scanners at the Foothills Medical Centre. Imaging was not available for one participant with stroke.

MRIcron software was used to delineate stroke lesions on either the T2-FLAIR or non-contrast CT (https://www.nitrc.org/projects/mricron) [61]. DWI and ADC scans were used to identify areas of acute ischemia and SWI and GRE were used to delineate the tissues damaged by intracranial hemorrhage [46]. A volume of interest (VOI) was obtained for each participant which contained the regions affected by stroke. The accuracy of the VOIs was verified by a neurologist blinded to the purpose and results of the study. Images were then registered to Montreal Neurological Institute (MNI) space using the Clinical Toolbox [62] in SPM12 [63] and the spm152 template in MRIcroGL (https://www.mccauslandcenter.sc.edu/mricrogl/) [61]. Distortion and warping of the damaged brain regions was avoided by applying cost function masks during image registration [64]. VOIs were visually inspected and compared to the original images to verify the accuracy of the registration process. Lesion overlap maps were generated in MRIcroGL to characterize the range and prevalence of lesions contained in the sample of stroke survivors [23, 24, 26].

### Clinical assessments

Participants underwent clinical assessment at a median of three days [range = 0–11] from when they performed the robotic tasks. The following assessments were administered: Medical Research Council—strength score composite (MRC; range of possible scores = 0–45, strength of shoulder flexion, extension, abduction, internal rotation, external rotation, elbow flexion, extension, forearm supination, and pronation) [65], Fugl-Meyer Assessment of Motor Recovery—Upper Extremity Motor Assessment (FMA; range of possible scores = 0–66, measures motor impairment in the hand and arm) [66], Modified Ashworth Scale (MAS; range of possible scores = 0–4, assesses spasticity of the elbow flexors) [67], Thumb Localization Test (TLT; range of possible scores = 0–3, measures arm proprioceptive impairments) [68], conventional sub-tests of the Behavioral Inattention Test (BIT, screens for hemispatial neglect) [69], and Functional Independence Measure (FIM; range of possible scores = 18–126, measures independence in performing activities of daily living) [70].

### Statistical analysis

Age was compared across stroke and control samples using bootstrap hypothesis tests (two tailed). Chi-squared tests were used to compare the biological sex composition of the stroke and control samples (% male and female). Measures of adaptation (*Initial Adaptation*, *Final Adaptation*, and *Trials to Adapt*) were compared across controls and participants with stroke using bootstrap hypothesis tests (one-tailed) [71]. Note, all bootstrap tests were performed by resampling the data 99,999 times in agreement with recommendations for hypothesis testing [71]. Spearman’s correlations were performed to assess how measures of adaptation relate to overall performance and individual measures derived from the proprioception tasks. The analyses were performed separately for the APM and AMM tasks. We bootstrapped the correlation analysis by resampling 99,999 times with replacement to obtain confidence bounds on Spearman’s rho (effect size). Correlations were interpreted based on statistical significance (*p*-value) as well as the strength of association according to established guidelines [72, 73]. Exact effect sizes (rho), bootstrapped confidence intervals on effect sizes, and *p*-values are reported in corresponding figures.

Normative data were used to identify individuals with impairments in proprioception, adaptation, both, or no impairments for both the APM and AMM tasks. Fisher’s exact tests were used to examine the categorical relationship (i.e., statistical independence) between impairments in adaptation and impairments in proprioception identified in the APM task. The same procedure was used to examine the categorical relationship between impairments in adaptation and impairments identified in the AMM task. Correlations and Fisher’s exact tests were used to examine relationships between measures of adaptation and TLT scores.

Bonferroni-Holm methods were used to correct for multiple statistical tests [74]. The corrected *p*-values are presented throughout the text (*α* = 0.05). Analyses were performed using custom scripts developed in MATLAB 2021b (MathWorks, Natick, MA).

## Results

Data were collected for 48 participants with stroke and 40 healthy controls. Lesion characteristics for participants with stroke are presented in Fig. 1E. Demographic and clinical features of the sample are presented in Table 1. The groups were matched for overall age (bootstrap: difference = − 0.97 years, CI [− 5.49, 3.61], *p* = 0.693) and sex (Chi-squared: difference = 17.5%, CI [− 3.24%, 36.4%], *X*^*2*^ = 2.66, *p* = 0.101). Participants with stroke were tested a median of 42.5 days post-stroke (range = 3–1580 days) and 35 (72.9%) were within the first six months of stroke. APM data was not available for one participant and AMM data was not available for three participants due to scheduling constraints. Proprioception was assessed using the TLT in 45 participants with stroke. Proprioceptive impairments were observed in 21 participants with stroke based on the TLT (46.7%; TLT Score > 0).

**Table 1:** Demographics and clinical characteristics of controls and participants with stroke

Demographics	Control	Stroke
N =	40	48
Age	62 [41–77]	63.5 [27–88]
Sex (F)	22 (55%)	18 (38%)
Handedness (R)	34 (85%)	45 (94%)
Clinical measures		
More affected arm (Dominant)		27 (56%)
Stroke type (Ischemic)		41 (85%)
Lesion volume (mL)		11.08 [0.27–191.88]
Days from stroke to robotic assessment		42.5 [3–1580]
MRC arm strength composite (/45)^Ɨ^		44 [25–45]
FMA—contralesional arm (/66)^ƗƗ^		60 [18–66]
FMA—ipsilesional arm (/66)^ƗƗ^		66 [60–66]
MAS ([0,1,1 + ,2,3,4])^ƗƗ^		[24,11,5,4,1,0]
TLT ([0,1,2,3])^ƗƗ^		[3, 5, 13, 24]
BIT (/146)^ƗƗƗ^		142 [116–146]
FIM (/126)^ƗƗƗƗ^		118 [87–126]

Demographic and clinical measures are presented as median [range]. Medical Research Council Strength Assessment—Arm Strength Composite (MRC; normal = 45); Fugl-Meyer Assessment of Motor Recovery—Upper Extremity (FMA; normal = 66), Modified Ashworth Scale (MAS; normal = 0; scale = 0,1,1 + ,2,3,4), Behavioural Inattention Test (BIT; neglect ≤ 129), Thumb Localization Test (TLT; normal = 0, slight difficulty = 1, moderate difficulty = 2, severe difficulty = 3), and Functional Independence Measure (FIM; normal = 126). ^Ɨ^MRC was obtained for 44 participants, ^ƗƗ^FMA, MAS, and TLT scores were available for 45 participants, ^ƗƗƗ^BIT was available for 39 participants, and ^ƗƗƗƗ^FIM was obtained for 47 participants

### Representative participant behaviour

**Fig. 2:**
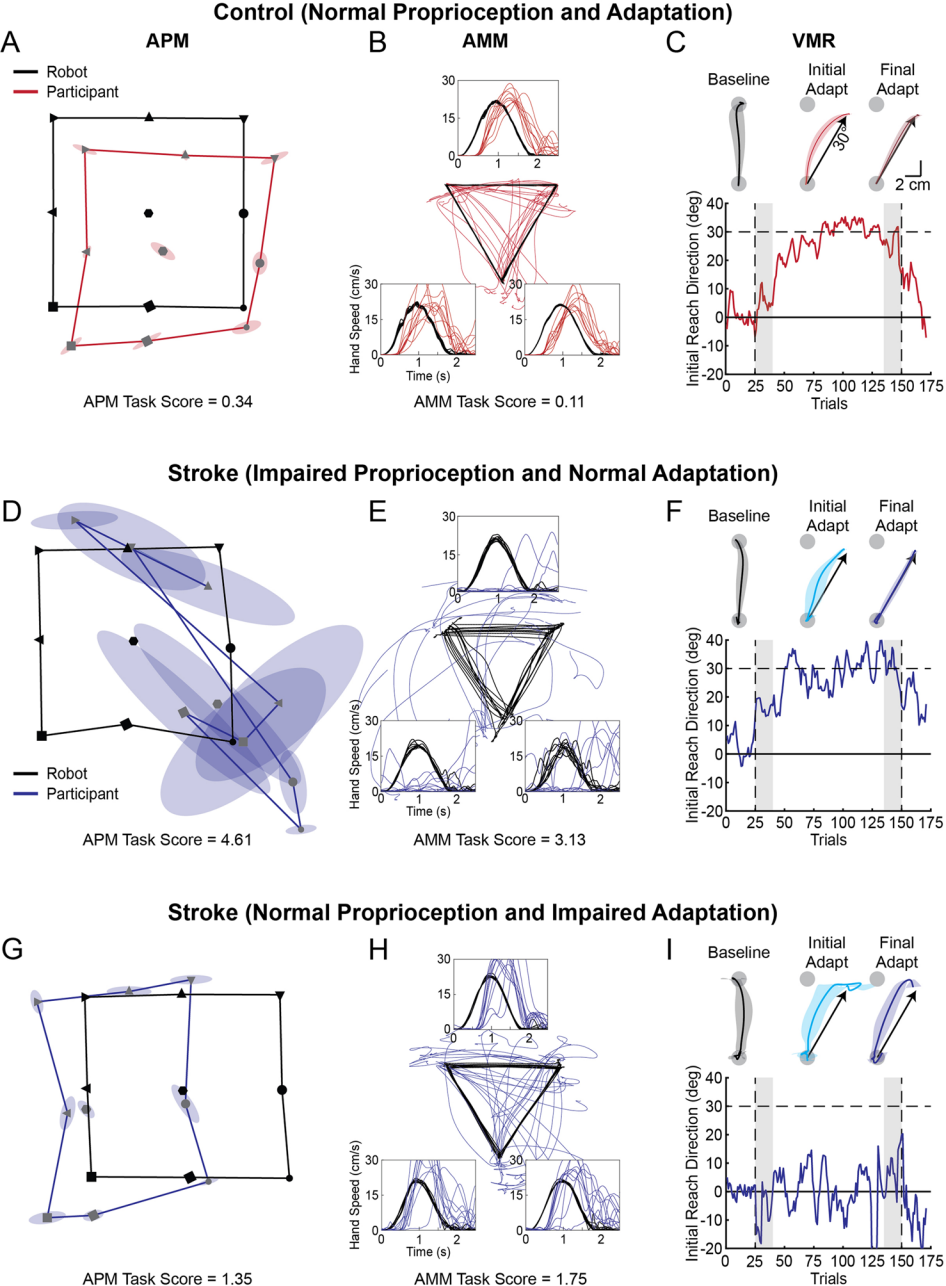
Exemplar arm position-matching (APM), arm movement-matching (AMM), and visuomotor adaptation (VMR) data for a control and two participants with stroke. **A** Representative control data. APM task. Average positions of the passive arm (moved by robot) are shown in black and are connected by black lines. The average matched positions of the active arm (moved by participant) are shown in red. Ellipses represent the variability of matching by the active arm at each position. Note that the matched positions of the active arm have been flipped about the participant’s midline to allow for visual comparison with the positions of the passive arm. **B **AMM task. Hand paths of the passive arm (moved by robot) and the hand speed profiles for each direction of movement are shown as black lines. The hand paths of the active arm (moved by participant) and corresponding hand speed profiles are shown in red. Note that the matched positions of the active arm have been flipped about the participant’s midline to allow for visual comparison with the hand paths of the passive arm. **C **VMR task. Average hand paths for Baseline, *Initial Adaptation*, and *Final Adaptation* (shaded regions = standard deviation) in the VMR task (arrow indicates 30° of adaptation—complete adaptation). The adaptation curve shows the trial-to-trial changes in the initial direction of the participant’s reaching movements during the VMR task. Grey shaded regions indicate the trials used to calculate *Initial* and *Final Adaptation*. **D–F** APM, AMM, and VMR data for a participant with stroke with impaired proprioception and normal adaptation. **G–I** APM, AMM, and VMR data for a participant with stroke with normal proprioception and impaired adaptation

Figure 2A–C show APM, AMM, and VMR data for a representative control. The control was consistent in matching the position of their arm in the APM task and did not contract or expand their workspace (Fig. 2A). They showed a small shift towards their body when matching the position of the robot. The amplitude of the spatial shift was well within the normal range for healthy adults. The control also responded promptly with their active arm in the AMM task (i.e., short *RL*) to match the peak speed, direction, and length of movement produced by the robot (Fig. 2B). The control participant displayed normal behaviour on the APM and AMM tasks (*Task Scores* < 1.96). During the baseline phase of the VMR task, the control made relatively straight reaching movements (Fig. 2C). In *Initial Adaptation*, the participant generated a rightward correction to counter the effects of the leftward (counterclockwise) rotation. They initiated movement to the right of the target after 20 trials (*Trials to Adapt*), and by *Final Adaptation*, were able to counter the effects of the 30° counterclockwise cursor rotation.

Figure 2D–F show a representative participant with stroke with impaired proprioception and normal adaptation. The participant made large errors in the APM task, showed a high degree of variability denoted by larger ellipses (Fig. 2D), and a systematic shift in their perceived workspace toward their midline. On the AMM task, they showed delayed responses, poor matching of the peak speed of their passive arm and made longer movements than the robot (Fig. 2E). The representative participant with stroke in Fig. 2F made relatively straight reaches in the baseline phase of the VMR task and made larger movement errors when the rotation was introduced in *Initial Adaptation*. They adapted to normal levels within 22 practice trials (*Trials to Adapt*) and countered the rotation by reaching nearly 30° clockwise in *Final Adaptation* (Fig. 2F).

The other representative participant with stroke performed within the normative range on the APM and AMM tasks but had impaired adaptation (Fig. 2G–I). They showed consistent matches in the APM task and a slight horizontal contraction of their workspace that remained within the normal range for healthy adults (*Task Score* < 1.96; Fig. 2G). In the AMM task, the participant matched the timing, path length, and initial direction of the movements made by the robot within the range of normal performance for healthy adults (Fig. 2H). Figure 2I shows that this participant demonstrated reduced levels of *Initial Adaptation* and *Final Adaptation* (less than 30°) and was unable to adapt to normal levels after 125 trials (*Trials to Adapt*).

### Performance on robotic tasks

Performance on the APM and AMM tasks was variable amongst participants with stroke (*APM Task Score*: mean = 2.10, range = [0.200–5.22]; *AMM Task Score*: mean = 1.94, range = [0.22–4.99]). Roughly half of the sample was impaired on the APM task (n = 24, 51.1%; *APM Task Score* > 1.96). Within our stroke sample, 17 (36.2%) participants with stroke had impairments on *AE*_*xy*_, 19 (40.4%) were impaired on *Var*_*xy*_, 20 (42.6%) were impaired on *Area*_*xy*_, and 11 (23.4%) were impaired on *Shift*_*xy*_. A large proportion of the stroke sample had impairments in the AMM task (n = 21, 44.4%, *AMM Task Score* > 1.96). Impairments in *RL* were observed in 10 (22.2%) participants with stroke, *SPR* was impaired in 12 (26.7%), *IDE* was impaired in 18 (40.0%), and *PLR* was impaired in 17 (37.7%) participants with stroke. Overall, impairments in *APM Task Score*, *AMM Task Score*, or both were observed in 28 (58.3%) participants with stroke.

**Fig. 3:**
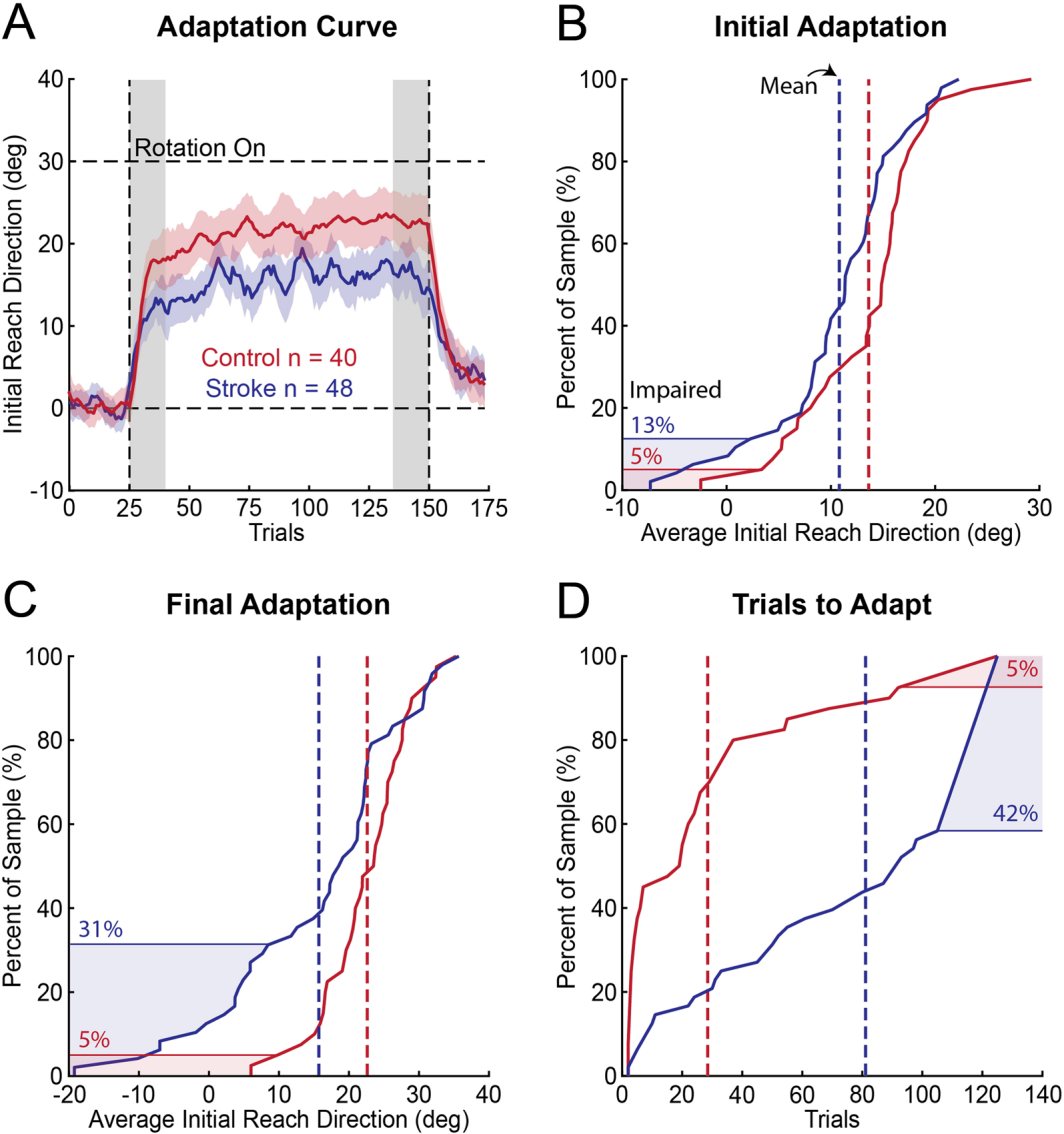
Average adaptation curves and empirical cumulative distribution functions describing the proportion of participants impaired on different measures of adaptation. **A** Group adaptation curves for controls (red) and participants with stroke (blue). The mean adaptation data (solid line) were smoothed using a moving average filter (window length = 5; overlap = 4). Shaded regions surrounding the mean represent the standard error. Gray regions indicate the trials used to calculate *Initial* and *Final Adaptation*. **B** Empirical cumulative distribution function for *Initial Adaptation*. Dashed lines indicate the mean amount of *Initial Adaptation*. Shaded regions indicate the proportion of individuals who failed *Initial Adaptation* (scored outside 95% of the controls data). **C** Empirical cumulative distribution function for *Final Adaptation* and **(D)** *Trials to Adapt*. **(C)** and **(D)** are presented in the same format as **(B)**

The average initial reach direction did not differ significantly between controls and participants with stroke in the baseline phase of the VMR task (bootstrap: difference = − 0.360°, CI [− 1.26°, 0.543°], *p* = 0.220; Fig. 3A), but were more variable in participants with stroke (standard deviation; bootstrap: difference = 3.27°, CI [1.71°, 4.85°], *p* < 0.001). On average, *Initial Adaptation* was lower in participants with stroke than controls (bootstrap: difference = − 2.84°, CI [− 5.40°, − 0.308°], *p* = 0.0173; Fig. 3A) and 6 (12.5%) participants with stroke were impaired (Fig. 3B). *Final Adaptation* was also reduced amongst participants with stroke (bootstrap: difference = − 6.93°, CI [− 11.0°, − 2.98°], *p* = 0.00120; Fig. 3A) and 15 (31.3%) participants with stroke were impaired (Fig. 3C). Lastly, participants with stroke required more *Trials to Adapt* than controls (bootstrap: difference = − 52.6, CI [− 68.8, − 35.7], *p* < 0.001). Twenty (41.7%) participants with stroke displayed impairments in the *Trials to Adapt* measure (Fig. 3D). Overall, 25 (52.1%) participants with stroke were impaired on at least one measure of adaptation.

### Performance on the APM task does not correlate with adaptation

**Fig. 4:**
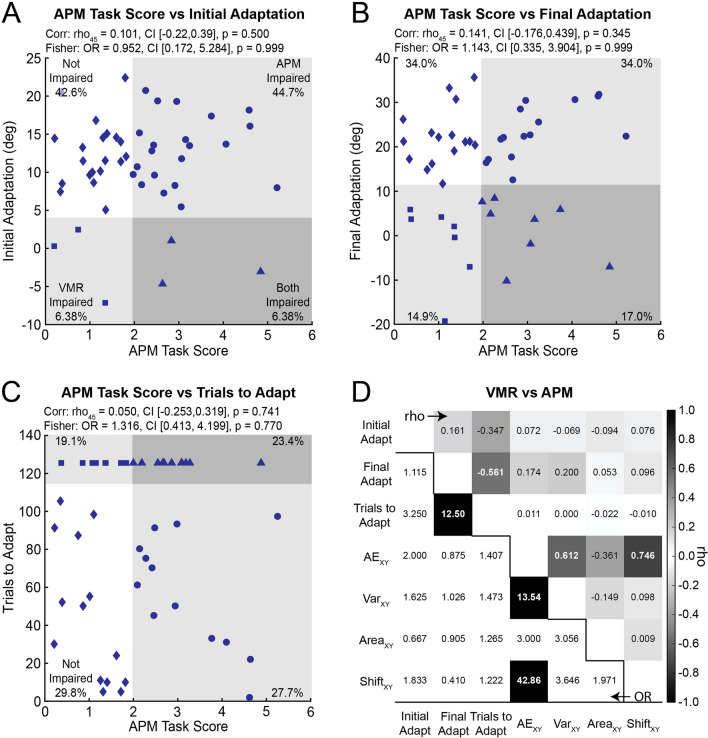
Relationship between the *APM Task Score* and **A** *Initial Adaptation*, **B** *Final Adaptation*, and **C** *Trials to Adapt* for participants with stroke. Symbols are used to highlight different impairment profiles: participants with normal limb-position sense and adaptation (solid diamond), participants with impaired adaptation (solid square), participants with impaired position sense (solid circle), and participants with impaired position sense and adaptation (solid triangle). Note that the *APM Task Score* was missing for one participant. **D** Spearman’s rho and Fisher’s exact tests of independence for the individual measures derived from the APM and VMR tasks. Upper right: Rho values from Spearman’s correlations. Bold white numbers indicate significant correlations between variables (*p* < 0.05 after Bonferroni-Holm corrections). Bottom left: Odds ratios from Fisher’s exact tests of independence. Black boxes with bold white numbers indicate significant categorical associations between variables (*p* < 0.05 after Bonferroni-Holm corrections)

We examined the relationship between performance on the APM and VMR tasks using Spearman’s correlations. Correlations between *APM Task Scores* and measures of adaptation were non-significant with weak effect sizes (Fig. 4A–C). Correlations between individual measures derived from the APM and VMR tasks were also non-significant with very weak effect sizes (Fig. 4D).

### Impairments in position sense are independent from impairments in adaptation

Next, we investigated if impairments in the APM and VMR tasks were statistically independent. We characterized impairment profiles by identifying individuals with impairments on the APM task, VMR task, both tasks, or neither task (Fig. 4). Note the proportion of participants with different profiles of impairment in the APM and VMR tasks are displayed in each quadrant of Fig. 4A–C. We tested the categorical relationship (i.e., statistical independence) between impairments in *APM Task Scores* and measures of adaptation using Fisher’s exact tests of independence. The results show that impairments in *APM Task Scores* were independent from impairments in *Initial Adaptation* (Fig. 4A), *Final Adaptation* (Fig. 4B), and *Trials to Adapt* (Fig. 4C). We also investigated how impairments on individual measures derived from the APM task related to impairments in measures from the VMR task. Fisher’s exact tests did not reveal significant categorical relationships between impairments on individual measures from the APM and VMR tasks (Fig. 4D).

### Performance on the AMM task correlates weakly with initial adaptation

**Fig. 5:**
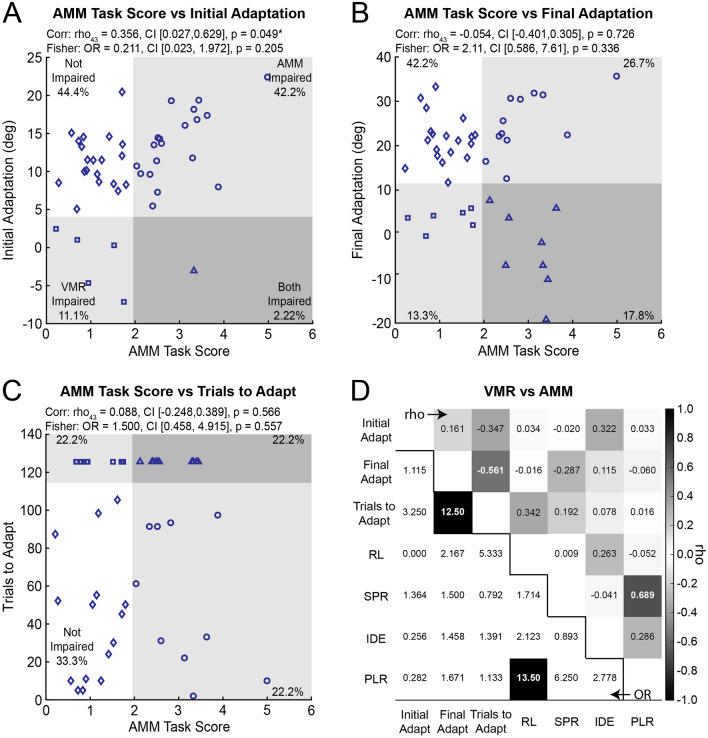
Relationship between the *AMM Task Score* and **A** *Initial Adaptation*, **B** *Final Adaptation*, and **C** *Trials to Adapt*. Symbols denote four unique impairment profiles: participants with normal kinesthetic sense and adaptation (open diamond), participants with impaired adaptation (open square), participants with impaired kinesthetic sense (open circle), and participants with impaired kinesthetic sense and adaptation (open triangle). Note that *AMM Task Scores* were not available for three participants. **D** Spearman’s correlations and Fisher’s exact tests of independence for the individual measures derived from the AMM and VMR tasks. Upper right: Rho values from Spearman’s correlations. Darker boxes indicate stronger correlations. Bold white numbers indicate significant correlations between variables (*p* < 0.05 after Bonferroni-Holm corrections). Bottom left: Odds ratios from the Fisher’s exact tests of independence. Black boxes with bold white numbers indicate significant categorical associations between variables (*p* < 0.05 after Bonferroni-Holm corrections)

Figure 5 shows scatterplots of the relationship between performance on the AMM task and measures of adaptation. We observed a weak positive correlation between *AMM Task Score* and *Initial Adaptation* suggesting that participants with more impaired kinesthetic sense may have adapted more in *Initial Adaptation* (Fig. 5A). In contrast, the correlations between *AMM Task Score*, *Final Adaptation* and *Trials to Adapt* were non-significant with very weak effect sizes (Fig. 5B and C). Correlations between individual measures derived from the AMM and VMR tasks were also non-significant with weak effect sizes (Fig. 5D).

### Kinesthetic impairments are independent from impairments in adaptation

The proportion of participants with different profiles of impairment in the AMM and VMR tasks are displayed in each quadrant of Fig. 5A–C. Fisher’s exact tests of independence revealed that impairments in *AMM Task Scores* were independent from impairments in *Initial Adaptation* (Fig. 5A), *Final Adaptation* (Fig. 5B), and *Trials to Adapt* in our sample (Fig. 5C). We also examined how impairments in individual measures from the AMM task related to measures from the VMR task. Fisher’s exact tests revealed impairments on measures derived from the AMM task were statistically independent of impairments in adaptation (Fig. 5D).

### Control analyses

There are a number of factors that may influence the potential relationship between and occurrence of impairments in proprioception and visuomotor adaptation. In separate analyses, we used Spearman’s partial correlations to examine the relationships between APM, AMM and VMR tasks while accounting for time post stroke, side of the more-affected arm (dominant vs. non-dominant), or spasticity measured by the Modified Ashworth Scale (MAS) as a covariate. We used logistic regression to examine the categorical relationship between impairments in the APM or AMM tasks with impairments in the VMR task while accounting for time post-stroke, the side of the more-affected arm, or MAS scores. Independent models were constructed where impairment in each measure in the APM or AMM task was included as a predictor of impairment in each measure of adaptation while including time post-stroke, the side of the more-affected arm, or MAS scores as a covariate. The results did not change when accounting for time post stroke (Additional file 3), the side of the stroke-affected limb (dominant, non-dominant; Additional file 4) or spasticity (Additional file 5).

In a different set of analyses, we performed Spearman’s correlations and Fisher’s Exact tests with a subsample that included only participants without hemispatial neglect (n = 34, identified with BIT; (Additional file 6), participants without ipsilesional motor impairments (n = 37, identified with FMA; (Additional file 7), and participants without ipsilesional proprioceptive impairments (n = 36, identified with TLT; (Additional file 8). Taken together, the results suggest that the relationships between performance in the APM, AMM, and VMR tasks were generally weak and non-significant with independent impairments.

### Visuomotor adaptation and proprioception assessed by the TLT

**Fig. 6:**
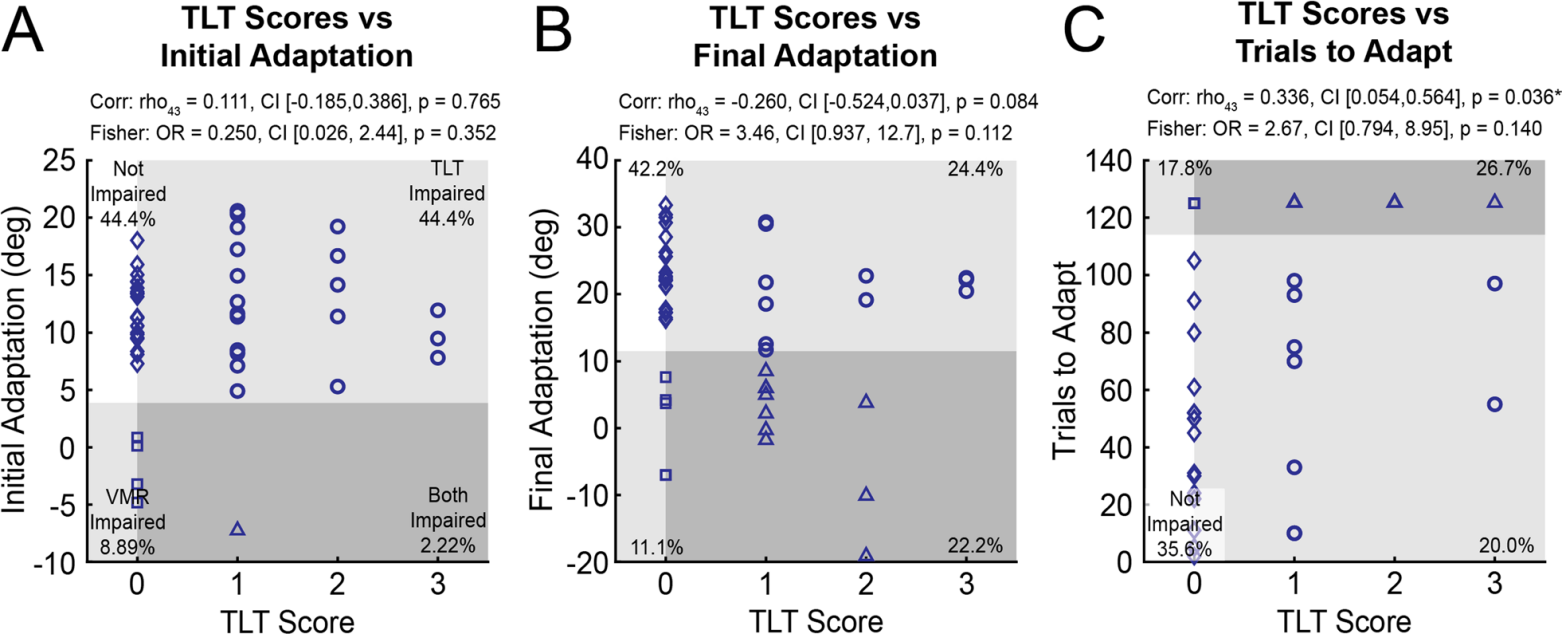
Thumb Localization Test (TLT) scores vs **A** *Initial Adaptation*, **B** *Final Adaptation*, and **C** *Trials to Adapt*. Four impairment profiles are shown: participants with normal proprioception (TLT = 0) and normal adaptation (open diamond), participants with impaired adaptation (open square), participants with impaired proprioception (TLT > 0; open circle), and participants with impaired proprioception and adaptation (open triangle). Spearman’s correlations and Fisher’s exact tests of independence are included. * indicates *p* < 0.05 after Bonferroni-Holm corrections

We recognize that robotic assessments of proprioceptive impairments are not commonplace in clinical rehabilitation settings and that past studies have used traditional observer-based ordinal scales [29]. We questioned how proprioception, assessed by the Thumb Localization Test (TLT), relates to visuomotor adaptation after stroke. Correlations between TLT scores and *Initial* and *Final Adaptation* were non-significant (Fig. 6A and B). We observed a weak correlation between TLT scores and *Trials to Adapt*, such that individuals with more severe proprioceptive impairments tended to require more *Trials to Adapt* (Fig. 6C). We also tested if proprioceptive impairments (TLT score > 0) were independent from impairments in visuomotor adaptation using Fisher’s exact tests. The analysis revealed that proprioceptive impairments assessed on the TLT were independent from impairments in adaptation (Fig. 6).

## Discussion

Proprioceptive impairments occur in 50–65% of individuals with stroke and are associated with worse motor recovery and rehabilitation outcomes [3]. Motor learning encompasses a range of processes that enable short- (i.e., motor adaptation) and long-term (i.e., motor skill learning) changes in motor behaviour [7]. Collectively, these processes are considered an important component of stroke rehabilitation [75], such that impairments in motor learning might also be associated with poor rehabilitation outcomes [76–78]. The relationship between impairments in proprioception and various components of motor learning is not well understood. Here, we used robotics to examine the relationship between position sense (APM task), kinesthetic sense (AMM task), and a task that assesses a specific type of motor learning known as visuomotor adaptation (VMR task) in 48 participants with stroke. We observed weak relationships between measures of performance on the APM task, AMM task, clinical TLT scores, and motor adaptation in the VMR task. Few of these relationships reached statistical significance. Impairments in proprioception, assessed using the APM task, AMM task and TLT, were independent from impairments in adaptation in our sample.

### Proprioceptive ability is weakly associated with visuomotor adaptation after stroke

There is ongoing debate about the role that proprioception plays in motor adaptation. Although some studies in healthy adults have reported a significant moderate association between position sense and visuomotor adaptation [31, 32], others studies have not observed such a relationship [33]. Our study adds a new perspective by examining the relationship between position sense and visuomotor adaptation in a sample of individuals recovering from stroke with a broad range of proprioceptive capabilities. Correlations between the APM and VMR tasks were non-significant with weak effect sizes, suggesting that position sense was not closely associated with the capacity to adapt movements to altered visual feedback. Our results are at odds with a previous study that reported a reduction in force-field adaptation amongst individuals with stroke who had reduced position sense [29]. The difference in results may be explained by methodological differences in the bimanual, robotic assessment of position sense in the current study versus clinical assessment of motion detection in the previous study. An alternative explanation is that visuomotor and force-field adaptation differ behaviourally [13, 34] and may rely on somewhat distinct neural substrates [35, 36], such that stroke may result in a range of distinct profiles of impairment in proprioception and forms of motor adaptation.

Some evidence suggests that spatial (position) and temporal (kinesthetic) aspects of proprioception may be processed differently and rely on distinct pathways in the nervous system [4, 37–39]. Position and kinesthetic sense can be differentially impaired with unique recovery profiles after stroke [4]. Thus, characterizing the relationship between kinesthetic sense and visuomotor adaptation adds to our understanding of the relationship between proprioception and motor adaptation after stroke. While past studies have focused primarily on position sense [31–33], one study in healthy adults did not find a relationship between kinesthetic sense and visuomotor adaptation [33], and another reported little change in kinesthetic sense following adaptation to a visuomotor rotation [79]. Our study is the first to show that kinesthetic sense and visuomotor adaptation are, at best, weakly associated in the post-stroke upper limb.

### Impairments in proprioception and adaptation are independent after stroke

Proprioceptive impairments assessed on the APM task, AMM task, and TLT were independent of impairments in adaptation. Naturally, this was related to the emergence of unique impairment profiles. While some individuals had impairments in proprioception and adaptation, others displayed normal adaptation despite having impaired proprioception. The latter finding suggests that proprioceptive impairments may not preclude normal adaptation in some individuals. This finding is interesting because it points towards the possibility that other factors may help to preserve adaptation. Past work has shown that some participants can use vision to compensate for proprioceptive impairments after stroke, while others cannot [47, 80]. It is possible that some participants with proprioceptive impairments may be able to rely more on visual error-feedback when adapting their movements [47, 81], though additional studies are required to test this idea.

A notable portion of our sample had normal proprioception but were impaired in adaptation. One interpretation is that impairments in adaptation may stem from other types of impairments in this group of participants. Studies in healthy adults implicate broad networks of motor, sensory, and cognitive areas of the brain in visuomotor adaptation [82–84]. A recent study reported that motor impairments were weak-moderately associated with reductions in visuomotor adaptation after stroke [26]. Together, the results point to the possibility that other factors, including visual or cognitive function [85, 86] may play into distinct profiles of impairment in motor adaptation in the early weeks and months after stroke. Some evidence in older adults suggest that reductions in visuomotor adaptation may be related to cognitive decline [87]. However, recent evidence in chronic stroke suggests that mild cognitive impairment may not account for impairments in visuomotor adaptation in the ipsilesional arm [88]. Larger studies are needed to explore the relationship between lesion location and adaptation as this may be useful for understanding how other types of impairments relate to impairments in visuomotor adaptation.

It is important to recognize that several factors can influence motor adaptation and proprioception after stroke. Previous reports have identified time post-stroke [26] and side of the stroke affected limb (i.e., dominant vs non-dominant) as factors that may influence visuomotor adaptation after stroke [23, 25]. Other studies have reported that proprioceptive function in the upper limb improves with time after stroke [1, 3, 46, 48, 89], and in healthy individuals, may also depend on limb dominance [48, 90, 91]. We repeated our analyses accounting for time post-stroke and side of the more-affected arm and found the overall findings were similar. Other studies have shown that hemispatial neglect may impact visuomotor adaptation [92]. Few participants in our sample presented with hemispatial neglect as identified with the Behavioural Inattention Test. We repeated our analysis including only participants without neglect and found similar results. Larger studies would better inform on the relationship between hemispatial neglect and visuomotor adaptation. A past study reported a weak and non-significant correlation between proprioceptive function, assessed with the APM task, and spasticity assessed with the Modified Ashworth Scale [93]. The lack of a significant relationship may reflect the fact that the robot moves the more affected arm with a bell-shaped speed profile that is relatively slow compared to the speeds required to trigger spasticity [94]. We repeated our analyses and found that associations between performance in the APM and visuomotor adaptation tasks changed very little when accounting for spasticity of the elbow flexors as a covariate (MAS scores). In separate analyses, we also verified these factors did not influence the independence of impairments in visuomotor adaptation and proprioception. Collectively, the results suggest the sense of limb position and motion are weakly associated with and are statistically independent from impairments in visuomotor adaptation.

### Measuring proprioception after stroke

There are a variety of tasks available to assess proprioception. These tasks fall into broad categories based on whether they assess proprioception using one or both arms or require participants to actively or passively establish the reference position or motion of the arm [51]. Unimanual tasks tend to produce smaller matching errors than bimanual tasks in healthy individuals. Errors also tend to be smaller in active tasks, in which participants first move their arm to the proprioceptive target before attempting to match, compared to tasks where the arm is passively moved by a robotic device or experimenter. There are several considerations when implementing these tasks in clinical populations. Tasks that require the participant to remember the location of a proprioceptive target can be problematic for patients with memory impairments. Active, unimanual tasks, on the other hand, can be confounded by motor impairment in the more-affected arm. Collectively, these factors can make it difficult to know if impairments in proprioception arise from memory, sensory, and/or motor impairments.

We chose bimanual tasks that assess the sense of limb position (APM) and motion (AMM). They involve matching the position or motion of the more-affected, passively-moved arm (contralesional) with the arm that is less affected by stroke (ipsilesional). These tasks eliminate the need to move the more-affected arm and do not require the participant to remember the location of the proprioceptive target. They do however, require the integration of proprioceptive information across cerebral hemispheres [95]. It is possible that lesions impacting structures involved in interhemispheric communication, like the corpus callosum, could impact performance on the APM and AMM tasks [49]. Ipsilesional motor impairments can also occur early after unilateral stroke (~ 37–47%) [96, 97] and may contribute to impairments identified in bimanual proprioception tasks. Ipsilesional proprioceptive impairments have been documented in unilateral tasks that require identifying when the position of the passively moved index and middle fingers are matched [98]. Although the prevalence of ipsilesional proprioceptive impairments in the arm is yet to be determined, they would in principle make it difficult to ascertain if impaired performance arises from impairments in the ipsilesional, contralesional, or both arms [2, 45]. In short, uni- [95] and bimanual tasks [2, 41] have been used to probe upper limb proprioceptive function in independent stroke samples, raising the question of whether they capture similar or distinct aspects of proprioceptive function.

We repeated our analyses after removing a small subset of the sample who presented with ipsilesional motor (identified with the FMA) or proprioceptive impairments (identified with the TLT) and found similar results. Additional studies that assess proprioception unilaterally and bilaterally in the same participants [33, 79, 99–101] may provide an alternative perspective of proprioceptive impairments and how they relate to motor adaptation after stroke.

### Implications for rehabilitation

Motor adaptation is a type of motor learning that involves short-term modifications in motor behaviour in response to changes in the body, environment, or demands of a task. Understanding how proprioceptive impairments relate to impairments in motor adaptation may broaden our general understanding of motor learning after stroke and could be helpful in the design and delivery of stroke rehabilitation. If motor adaptation and proprioceptive impairments are linked (i.e., dependent), then many individuals with proprioceptive impairments may also present with impairments in adaptation. In this case, treating an individual’s proprioceptive impairments might be a prerequisite to using rehabilitation strategies that rely on error-feedback to promote the adaptation of upper limb movements. Alternatively, new therapy approaches that accommodate for impairments in proprioception and motor adaptation may be required to promote motor recovery. In contrast, if impairments in proprioception and motor adaptation are independent, then it is possible that some individuals with impaired proprioception may still benefit from rehabilitation strategies that use error-feedback to adapt and improve the performance of upper limb movements through practice.

Therapists often instruct individuals with proprioceptive impairments to use vision to guide their limbs as they practice movements and motor skills [47, 80]. The approach assumes that individuals with proprioceptive impairments can process visual error-feedback and use it to modify their movements throughout practice [80]. This practice is supported by research in healthy adults showing that vision can enhance movement performance [102, 103], as well as studies showing that individuals with sensory deafferentation can adapt their reaching movements to a visuomotor rotation [81] or force field [104] at comparable rates to controls when visual feedback is provided. It is unclear how these findings apply to individuals with damage to sensory regions in the brain. One study examining a single case of chronic stroke has shown that providing visual feedback may improve the end-point accuracy of reaching movements in a participant with sensory impairments [105]. However, evidence from larger samples suggests that while some individuals with stroke can use vision to compensate for impairments in position and kinesthetic sense, many show no benefits in improving their movements through visual feedback [47, 80]. In our stroke sample we observed some participants with proprioceptive impairments that could adapt their reaching movements using visual error-feedback, and others who were unable. This suggests that using vision to help individuals with proprioceptive impairments adapt their movements may be a viable option for only some individuals with stroke. Understanding how impairments in visuomotor adaptation influence the ways in which individuals adapt their movements or attain long-term changes in performance through learning using visual information could be important for the planning and delivery of rehabilitation. Answering this question will require longitudinal studies that look at the relationship between short-term forms of learning, such as adaptation, as well as skill learning attained through long-term practice and retention.

### Limitations and future directions

Visuomotor adaptation is thought to be a useful model for understanding how individuals modify their movements using error feedback, but is different from other forms of long-term skill learning that can also occur in a clinical setting. Studies examining how visuomotor adaptation relates to other types of motor learning after stroke could be important for understanding the role of adaptation in stroke rehabilitation, as well as how various components of motor learning can be impaired after stroke. Longitudinal studies would also provide a better understanding of how impairments in motor adaptation evolve during stroke recovery [1].

Our study assessed visuomotor adaptation with continuous visual feedback of the position and motion of a cursor representing the participant’s hand. This is a common research practice [23–26], and is also representative of the ways in which individuals perform exercises and tasks in everyday living and rehabilitation settings. In contrast, the APM and AMM tasks were performed in the absence of vision to assess proprioception [2, 41]. Previous research has shown that visual feedback can help some individuals with stroke compensate for their proprioceptive impairments [47, 80], and can help individuals with sensory deafferentation adapt to a visuomotor rotation at a similar rate as controls [81]. Additional research is needed to explore how different types of visual feedback (e.g., continuous, endpoint, or no feedback) influence impairments in motor adaptation after stroke.

## Conclusions

Our data suggest that proprioception and visuomotor adaptation are weakly associated and that impairments in proprioception and visuomotor adaptation are independent after stroke. The results add to the growing debate over the role of proprioception in motor adaptation and raise questions about how other types of impairments and clinical variables relate to impairments in motor adaptation after stroke. Knowing how impairments in proprioception influence visuomotor adaptation adds to our broader understanding of motor learning after stroke and could be important for the design and delivery of stroke rehabilitation.

### Supplementary Information

**Additional file 1.** APM Measures.

**Additional file 2.** AMM Measures.

**Additional file 3.** Time Post Stroke.

**Additional file 4.** Side of More-Affected Arm.

**Additional file 5.** Modified Ashworth Scale.

**Additional file 6.** Neglect Excluded.

**Additional file 7.** Ipsilesional Motor Impairments Excluded.

**Additional file 8.** Ipsilesional Proprioceptive Impairments Excluded.

## Data Availability

The data used in the current study are not publicly available. Data may be made available from the corresponding author upon reasonable request.

## Abbreviations

ADC: Apparent diffusion coefficient
Area_xy_: Contraction/expansion
CT: Computed tomography
DWI: Diffusion-weighted imaging
AE_xy_: Absolute error
FIM: Functional independence measure
FLAIR: T2 fluid-attenuated inversion recovery
FM: Fugl-Meyer assessment of motor recovery—upper extremity motor assessment
GRE: Gradient echo
IDE: Initial direction error
MAS: Modified Ashworth Scale
AMM: Arm movement-matching
MNI: Montreal neurological institute
MRC: Medical research council strength score composite
MRI: Magnetic resonance imaging
PLR: Path length ratio
APM: Arm position-matching
PSR: Peak speed ratio
RL: Response latency
RSS: Root sum square
Shift_xy_: Spatial shift
SWI: Susceptibility-weighted imaging
TLT: Thumb localization test
Var_xy_: Variability
VMR: Visuomotor rotation
VOI: Volume of interest
