# Supplementary material for: The independence of impairments in proprioception and visuomotor adaptation after stroke
Source: J Neuroeng Rehabil. 2024 May 18;21:81. doi: 10.1186/s12984-024-01360-7 (PMC11102216; doi:10.1186/s12984-024-01360-7)
Supplement: Supplementary file 1 — Additional file 1. APM Measures. [file 12984_2024_1360_MOESM1_ESM.docx]

|  | |
| --- | --- |
| ***APM* Task Measures** | **Description** |
| **Absolute Error_x_** | The average absolute distance error in the x-axis across all targets. |
| **Absolute Error_y_** | The average absolute distance error in the y-axis across all targets. |
| **Variability_x_** | The standard deviation (SD) of the matched position in the x-axis for each target. SD were averaged across all targets. |
| **Variability_y_** | The standard deviation (SD) of the matched position in the y-axis for each target. SD were averaged across all targets. |
| **Spatial Area_x_** | The ratio of the range of movements in the x-axis made by the active arm relative to the range of movements in the x-axis made by the passive arm (robot moved). |
| **Spatial Area_y_** | The ratio of the range of movements in the y-axis made by the active arm relative to the range of movements in the y-axis made by the passive arm (robot moved). |
| **Spatial Shift_x_** | The average difference between mirrored x-positions of the active arm and the x-position of the passive arm. (+ = lateral shift; - = medial shift). |
| **Spatial Shift_y_** | The average difference between mirrored y-positions of the active arm and the y-position of the passive arm. (+ = distal shift; - = proximal shift). |

**Supplementary Materials 1.** Description of the measures used in *APM Task Score.*
