# Supplementary material for: The independence of impairments in proprioception and visuomotor adaptation after stroke
Source: J Neuroeng Rehabil. 2024 May 18;21:81. doi: 10.1186/s12984-024-01360-7 (PMC11102216; doi:10.1186/s12984-024-01360-7)
Supplement: Supplementary file 2 — Additional file 2. AMM Measures. [file 12984_2024_1360_MOESM2_ESM.docx]

|  | |
| --- | --- |
| ***AMM* Task Measures** | **Description** |
| **Failed Trials** | Number of failed trials. Participants failed a trial if any of the following occurred:   1. Active arm never exceeds 20% of the maximum speed of the passive arm. 2. The difference in time to movement onset between arms is greater than 3s. 3. If the onset or offset of movement is less than 350ms |
| **Response Latency** | Difference between time to movement onset for the active and passive arms averaged across all trials. |
| **Peak Speed Ratio** | The ratio between the peak speeds of the passive arm compared to the active arm averaged across all trials. |
| **Initial Direction Error** | The difference in initial direction of the movement (measured as peak hand velocity) for the passive and active arms averaged across all trials. |
| **Path Length Ratio** | The ratio of the distance of movement in the passive and active arms averaged across all trials |

**Supplementary Materials 2.** Description of the measures included in the *AMM Task Score*.
