# Supplementary material for: The independence of impairments in proprioception and visuomotor adaptation after stroke
Source: J Neuroeng Rehabil. 2024 May 18;21:81. doi: 10.1186/s12984-024-01360-7 (PMC11102216; doi:10.1186/s12984-024-01360-7)
Supplement: Supplementary file 3 — Additional file 3. Time Post Stroke. [file 12984_2024_1360_MOESM3_ESM.docx]

**Table 1. VMR vs APM Score with Time Post Stroke as a Covariate**

| **N = 47** | **Initial Adaptation** | **Final Adaptation** | **Trials to Adapt** |
| --- | --- | --- | --- |
| **APM Score**  **(Spearman correlation)** | rho_p_ = 0.175 (*p* = 0.257) | rho_p_ = 0.073 (*p* = 0.638) | rho_p_ = 0.036 (*p* = 0.814) |
| **APM Score**  **(Logistic regression model p-value)** | *p* = 0.448 | *p* = 0.852 | *p* = 0.370 |

Note: p-values are Bonferonni-Holm corrected.

**Table 2. VMR vs Individual APM Variables with Time Post Stroke as a Covariate**

| **N = 48** | **Initial Adaptation** | **Final Adaptation** | **Trials to Adapt** | **AE XY** | **Var XY** | **Area XY** | **Shift XY** |
| --- | --- | --- | --- | --- | --- | --- | --- |
| **Initial Adapt** |  | rho_p_ = 0.238 (*p* = 0.115) | rho_p_ = -0.392 (*p* = 0.140) | rho_p_ = 0.135 (*p* = 0.377) | rho_p_ = -0.004 (*p* = 0.980) | rho_p_ = -0.110 (*p* = 0.474) | rho_p_ = 0.128 (*p* = 0.404) |
| **Final Adapt** | *p* = 0.986 |  | rho_p_ = -0.592 (*p* < 0.001)* | rho_p_ = 0.111 (*p* = 0.469) | rho_p_ = 0.124 (*p* = 0.418) | rho_p_ = 0.044 (*p* = 0.775) | rho_p_ = 0.046 (*p* = 0.765) |
| **Trials to Adapt** | *p* = 0.196 | *p* = 0.015* |  | rho_p_ = -0.020 (*p* = 0.898) | rho_p_ = 0.037 (*p* = 0.808) | rho_p_ = 0.005 (*p* = 0.973) | rho_p_ = -0.047 (*p* = 0.759) |
| **AE XY** | *p* = 0.405 | *p* = 0.995 | *p* = 0.303 |  | rho_p_ = 0.597 (*p* < 0.001)* | rho_p_ = -0.347 (*p* = 0.330) | rho_p_ = 0.721. (*p* < 0.001)* |
| **Var XY** | *p* = 0.433 | *p* = 0.943 | *p* = 0.259 | *p* = 0.022* |  | rho_p_ = -0.140 (*p* = 0.360) | rho_p_ = 0.047 (*p* = 0.761) |
| **Area XY** | *p* = 0.399 | *p* = 0.945 | *p* = 0.369 | *p* = 0.133 | *p* = 0.095 |  | rho_p_ = -0.081 (*p* = 0.595) |
| **Shift XY** | *p* = 0.449 | *p* = 0.675 | *p* = 0.356 | *p* = 0.002* | *p* = 0.091 | *p* = 0.208 |  |

Note: p-values are Bonferonni-Holm corrected.

**Table 3. VMR vs AMM Score with Time Post Stroke as a Covariate**

| **N = 45** | **Initial Adaptation** | **Final Adaptation** | **Trials to Adapt** |
| --- | --- | --- | --- |
| **AMM Score**  **(Spearman correlation)** | rho_p_ = 0.387 (*p* = 0.034)* | rho_p_ = -0.046 (*p* = 0.770) | rho_p_ = 0.012 (*p* = 0.942) |
| **AMM Score**  **(Logistic regression model p-value)** | *p* = 0.115 | *p* = 0.517 | *p* = 0.138 |

Note: p-values are Bonferonni-Holm corrected.

**Table 4. VMR vs Individual AMM Variables with Time Post Stroke as a Covariate**

| **N = 47** | **Initial Adaptation** | **Final Adaptation** | **Trials to Adapt** | **RL** | **SPR** | **IDE** | **PLR** |
| --- | --- | --- | --- | --- | --- | --- | --- |
| **Initial Adapt** |  | rho_p_ = 0.238 (*p* = 0.115) | rho_p_ = -0.392 (*p* = 0.148) | rho_p_ = 0.099 (*p* = 0.523) | rho_p_ = -0.038 (*p* = 0.807) | rho_p_ = 0.363 (*p* = 0.262) | rho_p_ = 0.037 (*p* = 0.810) |
| **Final Adapt** | *p* = 0.986 |  | rho_p_ = -0.592 (*p* < 0.001)* | rho_p_ = -0.120 (*p* = 0.439) | rho_p_ = -0.290 (*p* = 0.057) | rho_p_ = 0.117 (*p* = 0.450) | rho_p_ = -0.042 (*p* = 0.787) |
| **Trials to Adapt** | *p* = 0.196 | *p* = 0.016* |  | rho_p_ = 0.349 (*p* = 0.327) | rho_p_ = 0.095 (*p* = 0.538) | rho_p_ = 0.036 (*p* = 0.815) | rho_p_ = -0.072 (*p* = 0.640) |
| **RL** | *p* = 0.055 | *p* = 0.779 | *p* = 0.635 |  | rho_p_ = -0.067 (*p* = 0.664) | rho_p_ = -0.364 (*p* = 0.271) | rho_p_ = -0.019 (*p* = 0.903) |
| **SPR** | *p* = 0.389 | *p* = 0.957 | *p* = 0.055 | *p* = 0.085 |  | rho_p_ = -0.143 (*p* = 0.354) | rho_p_ = 0.661 (*p* < 0.001)* |
| **IDE** | *p* = 0.213 | *p* = 0.850 | *p* = 0.163 | *p* = 0.530 | *p* = 0.837 |  | rho_p_ = 0.202 (*p* = 0.190) |
| **PLR** | *p* = 0.183 | *p* = 0.772 | *p* = 0.204 | *p* = 0.083 | *p* = 0.593 | *p* = 0.370 |  |

Note: p-values are Bonferonni-Holm corrected.

**Supplementary Materials 3:** Spearman’s correlations and logistic regression examining the relationships between measures of visuomotor adaptation and *APM Task Score* (**Table 1**), adaptation and measures derived from the APM task (**Table 2**), visuomotor adaptation and *AMM Task Score* (**Table 3**), and visuomotor adaptation and measures derived from the AMM task (**Table 4**) with time post-stroke included as a covariate.
