# Supplementary material for: The independence of impairments in proprioception and visuomotor adaptation after stroke
Source: J Neuroeng Rehabil. 2024 May 18;21:81. doi: 10.1186/s12984-024-01360-7 (PMC11102216; doi:10.1186/s12984-024-01360-7)
Supplement: Supplementary file 4 — Additional file 4. Side of More-Affected Arm. [file 12984_2024_1360_MOESM4_ESM.docx]

**Table 1. VMR vs APM Score with Dominant Arm Impaired as a Covariate**

| **N = 47** | **Initial Adaptation** | **Final Adaptation** | **Trials to Adapt** |
| --- | --- | --- | --- |
| **APM Score**  **(Spearman correlation)** | rho_p_ = 0.116 (*p* = 0.444) | rho_p_ = 0.062 (*p* = 0.685) | rho_p_ = 0.088 (*p* = 0.561) |
| **APM Score**  **(Logistic regression model p-value)** | *p* = 0.958 | *p* = 0.019* | *p* = 0.428 |

Note: p-values are Bonferonni-Holm corrected.

**Table 2. VMR vs Individual APM Variables with Dominant Arm Impaired as a Covariate**

| **N = 48** | **Initial Adaptation** | **Final Adaptation** | **Trials to Adapt** | **AE XY** | **Var XY** | **Area XY** | **Shift XY** |
| --- | --- | --- | --- | --- | --- | --- | --- |
| **Initial Adapt** |  | rho_p_ = 0.199 (*p* = 0.180) | rho_p_ = -0.358 (*p* = 0.244) | rho_p_ = 0.085 (*p* = 0.570) | rho_p_ = -0.056 (*p* = 0.709) | rho_p_ = -0.090 (*p* = 0.547) | rho_p_ = 0.077 (*p* = 0.608) |
| **Final Adapt** | *p* = 0.168 |  | rho_p_ = -0.560 (*p* < 0.001)* | rho_p_ = 0.101 (*p* = 0.501) | rho_p_ = 0.091 (*p* = 0.542) | rho_p_ = 0.023 (*p* = 0.876) | rho_p_ = 0.095 (*p* = 0.524) |
| **Trials to Adapt** | *p* = 0.222 | *p* = 0.017* |  | rho_p_ = 0.040 (*p* = 0.788) | rho_p_ = 0.041 (*p* = 0.786) | rho_p_ = -0.012 (*p* = 0.936) | rho_p_ = -0.007 (*p* = 0.962) |
| **AE XY** | *p* = 0.718 | *p* = 0.161 | *p* = 0.451 |  | rho_p_ = 0.589 (*p* < 0.001)* | rho_p_ = -0.387 (*p* = 0.129) | rho_p_ = 0.759 (*p* < 0.001)* |
| **Var XY** | *p* = 0.840 | *p* = 0.134 | *p* = 0.380 | *p* = 0.004* |  | rho_p_ = -0.181 (*p* = 0.223) | rho_p_ = 0.096 (*p* = 0.521) |
| **Area XY** | *p* = 0.842 | *p* = 0.151 | *p* = 0.511 | *p* = 0.179 | *p* = 0.172 |  | rho_p_ = -0.130 (*p* = 0.384) |
| **Shift XY** | *p* = 0.796 | *p* = 0.138 | *p* = 0.534 | *p* = 0.001* | *p* = 0.168 | *p* = 0.475 |  |

Note: p-values are Bonferonni-Holm corrected.

**Table 3. VMR vs AMM Score with Dominant Arm Impaired as a Covariate**

| **N = 45** | **Initial Adaptation** | **Final Adaptation** | **Trials to Adapt** |
| --- | --- | --- | --- |
| **AMM Score**  **(Spearman correlation)** | rho_p_ = 0.351 (*p* = 0.059) | rho_p_ = 0.009 (*p* = 0.954) | rho_p_ = 0.070 (*p* = 0.651) |
| **AMM Score**  **(Logistic Regression model p-value)** | *p* = 0.285 | *p* = 0.045* | *p* = 0.553 |

Note: p-values are Bonferonni-Holm corrected.

**Table 4. VMR vs Individual AMM Variables with Dominant Arm Impaired as a Covariate**

| **N = 48** | **Initial Adaptation** | **Final Adaptation** | **Trials to Adapt** | **RL** | **SPR** | **IDE** | **PLR** |
| --- | --- | --- | --- | --- | --- | --- | --- |
| **Initial Adapt** |  | rho_p_ = 0.199 (*p* = 0.180) | rho_p_ = -0.358 (*p* = 0.244) | rho_p_ = 0.052 (*p* = 0.733) | rho_p_ = -0.0414 (*p* = 0.785) | rho_p_ = 0.338 (*p* = 0.367 | rho_p_ = 0.031 (*p* = 0.839) |
| **Final Adapt** | *p* = 0.179 |  | rho_p_ = -0.560 (*p* < 0.001)* | rho_p_ = -0.115 (*p* = 0.449) | rho_p_ = -0.209 (*p* = 0.163) | rho_p_ = 0.062 (*p* = 0.682) | rho_p_ = -0.055 (*p* = 0.718) |
| **Trials to Adapt** | *p* = 0.222 | *p* = 0.019* |  | rho_p_ = 0.379 (*p* = 0.179) | rho_p_ = 0.167 (*p* = 0.267) | rho_p_ = 0.098 (*p* = 0.517) | rho_p_ = 0.013 (*p* = 0.932) |
| **RL** | *p* = 0.160 | *p* = 0.118 | *p* = 0.508 |  | rho_p_ = 0.064 (*p* = 0.674) | rho_p_ = 0.241 (*p* = 0.107) | rho_p_ = -0.048 (*p* = 0.754) |
| **SPR** | *p* = 0.857 | *p* = 0.213 | *p* = 0.588 | *p* = 0.442 |  | rho_p_ = -0.006 (*p* = 0.969) | rho_p_ = 0.705 (*p* < 0.001)* |
| **IDE** | *p* = 0.379 | *p* = 0.190 | *p* = 0.572 | *p* = 0.555 | *p* = 0.983 |  | rho_p_ = 0.292 (*p* = 0.780) |
| **PLR** | *p* = 0.427 | *p* = 0.162 | *p* = 0.646 | *p* = 0.059 | *p* = 0.305 | *p* = 0.244 |  |

Note: p-values are Bonferonni-Holm corrected.

**Supplementary Materials 4:** Spearman’s correlations and logistic regression examining the relationships between measures of visuomotor adaptation and *APM Task Score* (**Table 1**), visuomotor adaptation and measures derived from the APM task (**Table 2**), visuomotor adaptation and *AMM Task Score* (**Table 3**), and visuomotor adaptation and measures derived from the AMM task (**Table 4**) with side of the more-affected limb (dominant vs non-dominant) included as a covariate.
