# Supplementary material for: The independence of impairments in proprioception and visuomotor adaptation after stroke
Source: J Neuroeng Rehabil. 2024 May 18;21:81. doi: 10.1186/s12984-024-01360-7 (PMC11102216; doi:10.1186/s12984-024-01360-7)
Supplement: Supplementary file 5 — Additional file 5. Modified Ashworth Scale. [file 12984_2024_1360_MOESM5_ESM.docx]

**Table 1. VMR vs APM Score with Modified Ashworth Scale (MAS) as a Covariate**

| **N = 44** | **Initial Adaptation** | **Final Adaptation** | **Trials to Adapt** |
| --- | --- | --- | --- |
| **APM Score**  **(Spearman correlation)** | rho_p_ = 0.110 (*p* = 0.482) | rho_p_ = 0.166 (*p* = 0.286) | rho_p_ = 0.032 (*p* = 0.839) |
| **APM Score**  **(Logistic Regression model p-value)** | *p* = 0.957 | *p* = 0.091 | *p* = 0.345 |

Note: p-values are Bonferonni-Holm corrected.

**Table 2. VMR vs Individual APM Variables with Modified Ashworth Scale (MAS) as a Covariate**

| **N = 45** | **Initial Adaptation** | **Final Adaptation** | **Trials to Adapt** | **AE XY** | **Var XY** | **Area XY** | **Shift XY** |
| --- | --- | --- | --- | --- | --- | --- | --- |
| **Initial Adapt** |  | rho_p_ = 0.158 (*p* = 0.307) | rho_p_ = -0.396 (*p* = 0.140) | rho_p_ = 0.083 (*p* = 0.594) | rho_p_ = -0.099 (*p* = 0.523) | rho_p_ = -0.115 (*p* = 0.459) | rho_p_ = 0.109 (*p* = 0.480) |
| **Final Adapt** | *p* = 0.922 |  | rho_p_ = -0.496 (*p* = 0.012)* | rho_p_ = 0.226 (*p* = 0.140) | rho_p_ = 0.170 (*p* = 0.271) | rho_p_ = 0.016 (*p* = 0.919) | rho_p_ = 0.202 (*p* = 0.190) |
| **Trials to Adapt** | *p* = 0.199 | *p* = 0.011* |  | rho_p_ = -0.020 (*p* = 0.896) | rho_p_ = 0.046 (*p* = 0.769) | rho_p_ = 0.012 (*p* = 0.936) | rho_p_ = -0.096 (*p* = 0.537) |
| **AE XY** | *p* = 0.561 | *p* = 0.074 | *p* = 0.306 |  | rho_p_ = 0.642 (*p* < 0.001)* | rho_p_ = -0.348 (*p* = 0.357) | rho_p_ = 0.754 (*p* < 0.001)* |
| **Var XY** | *p* = 0.632 | *p* = 0.083 | *p* = 0.239 | *p* = 0.005* |  | rho_p_ = -0.151 (*p* = 0.328) | rho_p_ = 0.143 (*p* = 0.353) |
| **Area XY** | *p* = 0.973 | *p* = 0.073 | *p* = 0.317 | *p* = 0.303 | *p* = 0.161 |  | rho_p_ = -0.118 (*p* = 0.447) |
| **Shift XY** | *p* = 0.695 | *p* = 0.284 | *p* = 0.312 | *p* = 0.001* | *p* = 0.085 | *p* = 0.468 |  |

Note: p-values are Bonferonni-Holm corrected.

**Table 3. VMR vs AMM Score with Modified Ashworth Scale (MAS) as a Covariate**

| **N = 42** | **Initial Adaptation** | **Final Adaptation** | **Trials to Adapt** |
| --- | --- | --- | --- |
| **AMM Score**  **(Spearman correlation)** | rho_p_ = 0.236 (*p* = 0.140) | rho_p_ = -0.084 (*p* = 0.601) | rho_p_ = 0.057 (*p* = 0.722) |
| **AMM Score**  **(Logistic regression model p-value)** | *p* = 0.511 | *p* = 0.106 | *p* = 0.198 |

Note: p-values are Bonferonni-Holm corrected.

**Table 4. VMR vs Individual AMM Variables with Modified Ashworth Scale (MAS) as a Covariate**

| **N = 44** | **Initial Adaptation** | **Final Adaptation** | **Trials to Adapt** | **RL** | **SPR** | **IDE** | **PLR** |
| --- | --- | --- | --- | --- | --- | --- | --- |
| **Initial Adapt** |  | rho_p_ = 0.158 (*p* = 0.307) | rho_p_ = -0.396 (*p* = 0.147) | rho_p_ = 0.010 (*p* = 0.947) | rho_p_ = -0.016 (*p* = 0.917) | rho_p_ = 0.237 (*p* = 0.127) | rho_p_ = 0.049 (*p* = 0.756) |
| **Final Adapt** | *p* = 0.922 |  | rho_p_ = -0.496 (*p* = 0.012)* | rho_p_ = 0.016 (*p* = 0.921) | rho_p_ = -0.257 (*p* = 0.096) | rho_p_ = 0.167 (*p* = 0.284) | rho_p_ = -0.009 (*p* = 0.954) |
| **Trials to Adapt** | *p* = 0.199 | *p* = 0.013* |  | rho_p_ = 0.338 (*p* = 0.483) | rho_p_ = 0.129 (*p* = 0.409) | rho_p_ = 0.025 (*p* = 0.874) | rho_p_ = -0.068 (*p* = 0.664) |
| **RL** | *p* = 0.213 | *p* = 0.077 | *p* = 0.051 |  | rho_p_ = -0.045 (*p* = 0.777) | rho_p_ = 0.266 (*p* = 0.085) | rho_p_ = -0.107 (*p* = 0.494) |
| **SPR** | *p* = 0.788 | *p* = 0.086 | *p* = 0.303 | *p* = 0.584 |  | rho_p_ = -0.043 (*p* = 0.783) | rho_p_ = 0.647 (*p* < 0.001)* |
| **IDE** | *p* = 0.489 | *p* = 0.085 | *p* = 0.333 | *p* = 0.068 | *p* = 0.074 |  | rho_p_ = 0.329 (*p* = 0.528) |
| **PLR** | *p* = 0.638 | *p* = 0.079 | *p* = 0.345 | *p* = 0.056 | *p* = 0.062 | *p* = 0.248 |  |

Note: p-values are Bonferonni-Holm corrected.

**Supplementary Materials 5:** Spearman’s correlations and logistic regression examining the relationships between measures of visuomotor adaptation and *APM Task Score* (**Table 1**), visuomotor adaptation and measures derived from the APM task (**Table 2**), *AMM Task Score* (**Table 3**), and measures derived from the AMM task (**Table 4**) with spasticity (assessed using the Modified Ashworth Scale) included as a covariate.
