# Supplementary material for: The independence of impairments in proprioception and visuomotor adaptation after stroke
Source: J Neuroeng Rehabil. 2024 May 18;21:81. doi: 10.1186/s12984-024-01360-7 (PMC11102216; doi:10.1186/s12984-024-01360-7)
Supplement: Supplementary file 6 — Additional file 6. Neglect Excluded. [file 12984_2024_1360_MOESM6_ESM.docx]

**Table 1. VMR vs APM Scores after Removing Participants with Neglect**

| **N = 34** | **Initial Adaptation** | **Final Adaptation** | **Trials to Adapt** |
| --- | --- | --- | --- |
| **APM Score**  **(Spearman correlation)** | rho = 0.274, (*p* = 0.116) | rho = -0.111(*p* = 0.531) | rho = 0.229 (*p* = 0.193) |
| **APM Score**  **(Fisher’s Exact test)** | OR = 1.61 (*p* = 1.00) | OR = 2.89 (*p* = 0.282) | OR = 2.06 (*p* = 0.491) |

Note: p-values are Bonferonni-Holm corrected.

**Table 2. VMR vs Individual APM Variables after Removing Participants with Neglect**

| **N = 35** | **Initial Adaptation** | **Final Adaptation** | **Trials to Adapt** | **AE XY** | **Var XY** | **Area XY** | **Shift XY** |
| --- | --- | --- | --- | --- | --- | --- | --- |
| **Initial Adapt** |  | rho = 0.132 (*p* = 0.451) | rho = -0.292 (*p* = 0.089) | rho = 0.207 (*p* = 0.232) | rho = -0.029 (*p* = 0.869) | rho = -0.100 (*p* = 0.568) | rho = 0.150 (*p* = 0.389) |
| **Final Adapt** | OR = 1.33 (*p* = 1.00) |  | rho = -0.504 (*p* = 0.038)* | rho = -0.057 (*p* = 0.744) | rho = -0.089 (*p* = 0.613) | rho = 0.284 (*p* = 0.098) | rho = 0.021 (*p* = 0.905) |
| **Trials to Adapt** | OR = 6.91. (*p* = 0.141) | OR = 8.50 (*p* = 0.203) |  | rho = 0.193 (*p* = 0.267) | rho = 0.210 (*p* = 0.227) | rho = -0.154 (*p* = 0.379) | rho = 0.090 (*p* = 0.606) |
| **AE XY** | OR = 3.50 (*p* = 0.313) | OR = 1.63 (*p* = 0.709) | OR = 2.63 (*p* = 0.283) |  | rho = 0.539 (*p* = 0.017)* | rho = -0.215 (*p* = 0.216) | rho = 0.729 (*p* < 0.001)* |
| **Var XY** | OR = 3.00 (*p* = 0.337) | OR = 2.29 (*p* = 0.293) | OR = 3.43 (*p* = 0.157) | OR = 14.3 (*p* = 0.040)* |  | rho = -0.004 (*p* = 0.983) | rho = 0.006. (*p* = 0.974) |
| **Area XY** | OR = 1.00 (*p* = 1.00) | OR = 1.11 (*p* = 1.00) | OR = 1.63 (*p* = 0.511) | OR = 3.20 (*p* = 0.153) | OR = 2.50 (*p* = 0.288) |  | rho = -0.039 (*p* = 0.823) |
| **Shift XY** | OR = 2.67 (*p* = 0.568) | OR = 0.567 (*p* = 0.686) | OR = 1.45 (*p* = 0.700) | OR = 30.8 (*p* = 0.017)* | OR = 3.96 (*p* = 0.116) | OR = 1.70 (*p* = 0.685) |  |

Note: p-values are Bonferonni-Holm corrected.

**Table 3. VMR vs AMM Scores after Removing Participants with Neglect**

| **N = 33** | **Initial Adaptation** | **Final Adaptation** | **Trials to Adapt** |
| --- | --- | --- | --- |
| **AMM Score**  **(Spearman correlation)** | rho = 0.191 (*p* = 0.287) | rho = -0.385 (*p* = 0.083) | rho = 0.291 (*p* = 0.101) |
| **AMM Score**  **(Fisher’s exact test)** | OR = 0.450 (*p* = 0.643) | OR = 7.88 (*p* = 0.053) | OR = 3.06 (*p* = 0.163) |

Note: p-values are Bonferonni-Holm corrected.

**Table 4. VMR vs Individual AMM Variables after Removing Participants with Neglect**

| **N = 34** | **Initial Adaptation** | **Final Adaptation** | **Trials to Adapt** | **RL** | **SPR** | **IDE** | **PLR** |
| --- | --- | --- | --- | --- | --- | --- | --- |
| **Initial Adapt** |  | rho = 0.132 (*p* = 0.451) | rho = -0.292 (*p* = 0.089) | rho = 0.111 (*p* = 0.534) | rho = 0.112 (*p* = 0.528) | rho = 0.248 (*p* = 0.157) | rho = 0.046 (*p* = 0.795) |
| **Final Adapt** | OR = 1.33 (*p* = 1.00) |  | rho = -0.504 (*p* = 0.040)* | rho = -0.137 (*p* = 0.441) | rho = -0.251 (*p* = 0.153) | rho = -0.088 (*p* = 0.620) | rho = -0.071 (*p* = 0.691) |
| **Trials to Adapt** | OR = 6.91 (*p* = 0.141) | OR = 8.50 (*p* = 0.214) |  | rho = 0.474 (*p* = 0.0873) | rho = 0.137 (*p* = 0.442) | rho = 0.242 (*p* = 0.167) | rho = -0.099 (*p* = 0.576) |
| **RL** | OR = 0.00 (*p* = 0.559) | OR = 5.00 (*p* = 0.154) | OR = 9.00 (*p* = 0.066) |  | rho = 0.133 (*p* = 0.453) | rho = 0.408 (*p* = 0.302) | rho = 0.031 (*p* = 0.862) |
| **SPR** | OR = 2.56 (*p* = 0.570) | OR = 2.25 (*p* = 0.410) | OR = 0.700 (*p* = 1.00) | OR = 0.600 (*p* = 1.00) |  | rho = -0.075 (*p* = 0.674) | rho = 0.655 (*p* < 0.001)* |
| **IDE** | OR = 0.475 (*p* = 1.00) | OR = 1.90 (*p* = 0.459) | OR = 1.08 (*p* = 1.00) | OR = 1.06 (*p* = 1.00) | OR = 0.630 (*p* = 1.00) |  | rho = 0.126 (*p* = 0.479) |
| **PLR** | OR = 0.475 (*p* = 1.00) | OR = 3.40 (*p* = 0.138) | OR = 1.87 (*p* = 0.475) | OR = 18.3 (*p* = 0.172) | OR = 2.71 (*p* = 0.388) | OR = 2.36 (*p* = 0.434) |  |

Note: p-values are Bonferonni-Holm corrected.

**Supplementary Materials 6:** Spearman’s correlations and Fisher’s exact tests examining the relationships between measures of visuomotor adaptation and *APM Task Score* (**Table 1**), visuomotor adaptation and measures derived from the APM task (**Table 2**), visuomotor adaptation and *AMM Task Score* (**Table 3**), and visuomotor adaptation and measures derived from the AMM task (**Table 4**) in a subsample of participants without hemispatial neglect (assessed using the Behavioural Inattention Test).
