# Supplementary material for: The independence of impairments in proprioception and visuomotor adaptation after stroke
Source: J Neuroeng Rehabil. 2024 May 18;21:81. doi: 10.1186/s12984-024-01360-7 (PMC11102216; doi:10.1186/s12984-024-01360-7)
Supplement: Supplementary file 7 — Additional file 7. Ipsilesional Motor Impairments Excluded. [file 12984_2024_1360_MOESM7_ESM.docx]

**Table 1. VMR vs APM Scores after Removing Participants with Ipsilesional Motor Impairments (FMA)**

| **N = 37** | **Initial Adaptation** | **Final Adaptation** | **Trials to Adapt** |
| --- | --- | --- | --- |
| **APM Score**  **(Spearman correlation)** | rho = 0.075, (*p* = 0.659) | rho = 0.126, (*p* = 0.456) | rho = 0.086, (*p* = 0.614) |
| **APM Score**  **(Fisher’s Exact test)** | OR = 3.19, (*p* = 0.604) | OR = 1.52, (*p* = 0.728) | OR = 1.75, (*p* = 0.515) |

Note: p-values are Bonferonni-Holm corrected.

**Table 2. VMR vs Individual APM Variables after Removing Participants with Ipsilesional Motor Impairments (FMA)**

| **N = 38** | **Initial Adaptation** | **Final Adaptation** | **Trials to Adapt** | **AE XY** | **Var XY** | **Area XY** | **Shift XY** |
| --- | --- | --- | --- | --- | --- | --- | --- |
| **Initial Adapt** |  | rho = 0.034, (*p* = 0.838) | rho = -0.258, (*p* = 0.117) | rho = 0.078, (*p* = 0.640) | rho = -0.089, (*p* = 0.595) | rho = -0.047, (*p* = 0.780) | rho = 0.138, (*p* = 0.408) |
| **Final Adapt** | OR = 0.697, (*p* = 1.00) |  | rho = -0.558, (*p* = 0.005)* | rho = 0.135, (*p* = 0.419) | rho = 0.271, (*p* = 0.100) | rho = 0.198, (*p* = 0.232) | rho = -0.013, (*p* = 0.940) |
| **Trials to Adapt** | OR = 4.29, (*p* = 0.307) | OR = 13.6, (*p* = 0.032)* |  | rho = 0.026, (*p* = 0.878) | rho = 0.007, (*p* = 0.966) | rho = -0.102, (*p* = 0.542) | rho = -0.044, (*p* = 0.791) |
| **AE XY** | OR = 7.20, (*p* = 0.107) | OR = 0.944, (*p* = 1.00) | OR = 1.75, (*p* = 0.502) |  | rho = 0.632, (*p* < 0.001)* | rho = -0.293, (*p* = 0.074) | rho = 0.718, (*p* < 0.001)* |
| **Var XY** | OR = 4.29, (*p* = 0.307) | OR = 0.833, (*p* = 1.00) | OR = 1.83, (*p* = 0.513) | OR = 17.4, (*p* = 0.011)* |  | rho = -0.088, (*p* = 0.598) | rho = 0.078, (*p* = 0.640) |
| **Area XY** | OR = 1.43, (*p* = 1.00) | OR = 0.974, (*p* = 1.00) | OR = 2.25, (*p* = 0.324) | OR = 2.07, (*p* = 0.323) | OR = 2.25, (*p* = 0.324) |  | rho = -0.047, (*p* = 0.778) |
| **Shift XY** | OR = 5.80, (*p* = 0.148) | OR = 0.840, (*p* = 1.00) | OR = 1.85, (*p* = 0.678) | OR = ∞, (*p* = 0.003)* | OR = 10.9, (*p* = 0.563) | OR = 2.11, (*p* = 0.425) |  |

Note: p-values are Bonferonni-Holm corrected.

**Table 3. VMR vs AMM Scores after Removing Participants with Ipsilesional Motor Impairments (FMA)**

| **N = 36** | **Initial Adaptation** | **Final Adaptation** | **Trials to Adapt** |
| --- | --- | --- | --- |
| **AMM Score**  **(Spearman correlation)** | rho = 0.359, (*p* = 0.094) | rho = -0.120, (*p* = 0.487) | rho = 0.116, (*p* = 0.501) |
| **AMM Score**  **(Fisher’s Exact test)** | OR = 0.429, (*p* = 0.626) | OR = 3.72, (*p* = 0.141) | OR = 2.44, (*p* = 0.311) |

Note: p-values are Bonferonni-Holm corrected.

**Table 4. VMR vs Individual AMM Variables after Removing Participants with Ipsilesional Motor Impairments (FMA)**

| **N = 37** | **Initial Adaptation** | **Final Adaptation** | **Trials to Adapt** | **RL** | **SPR** | **IDE** | **PLR** |
| --- | --- | --- | --- | --- | --- | --- | --- |
| **Initial Adapt** |  | rho = 0.034, (*p* = 0.838) | rho = -0.258, (*p* = 0.117) | rho = -0.055, (*p* = 0.744) | rho = -0.023, (*p* = 0.895) | rho = 0.328, (*p* = 0.907) | rho = 0.085, (*p* = 0.616) |
| **Final Adapt** | OR = 0.697, (*p* = 1.00) |  | rho = -0.558, (*p* = 0.005)* | rho = -0.017, (*p* = 0.920) | rho = -0.270, (*p* = 0.107) | rho = 0.079, (*p* = 0.640) | rho = -0.016, (*p* = 0.926) |
| **Trials to Adapt** | OR = 4.29, (*p* = 0.307) | OR = 13.6, (*p* = 0.0357)* |  | rho = 0.414, (*p* = 0.011) | rho = 0.230, (*p* = 0.213) | rho = 0.088, (*p* = 0.604) | rho = -0.037, (*p* = 0.828) |
| **RL** | OR = 0.00, (*p* = 0.554) | OR = 3.75, (*p* = 0.116) | OR = 6.30, (*p* = 0.052) |  | rho = -0.002, (*p* = 0.989) | rho = 0.296, (*p* = 0.075) | rho = -0.051, (*p* = 0.766) |
| **SPR** | OR = 1.24, (*p* = 1.00) | OR = 1.33, (*p* = 1.00) | OR = 0.643, (*p* = 0.701) | OR = 1.05, (*p* = 1.00) |  | rho = -0.107, (*p* = 0.529) | rho = 0.605, (*p* = 0.002)* |
| **IDE** | OR = 0.452, (*p* = 0.633) | OR = 1.78, (*p* = 0.488) | OR = 1.65, (*p* = 0.516) | OR = 2.25, (*p* = 0.438) | OR = 0.850, (*p* = 1.00) |  | rho = 0.219, (*p* = 0.193) |
| **PLR** | OR = 0.00, (*p* = 0.282) | OR = 1.84, (*p* = 0.468) | OR = 1.27, (*p* = 1.00) | OR = 16.1, (*p* = 0.040)* | OR = 2.63, (*p* = 0.395) | OR = 2.97, (*p* = 0.164) |  |

Note: p-values are Bonferonni-Holm corrected.

**Supplementary Materials 7:** Spearman’s correlations and Fisher’s exact tests examining the relationships between measures of visuomotor adaptation and *APM Task Score* (**Table 1**), visuomotor adaptation and measures derived from the APM task (**Table 2**), visuomotor adaptation and *AMM Task Score* (**Table 3**), and visuomotor adaptation and measures derived from the AMM task (**Table 4**) in a subsample of participants without ipsilesional motor impairments (assessed using the Fugl-Meyer Assessment of Motor Recovery – Upper Extremity).
