# Supplementary material for: The independence of impairments in proprioception and visuomotor adaptation after stroke
Source: J Neuroeng Rehabil. 2024 May 18;21:81. doi: 10.1186/s12984-024-01360-7 (PMC11102216; doi:10.1186/s12984-024-01360-7)
Supplement: Supplementary file 8 — Additional file 8. Ipsilesional Proprioceptive Impairments Excluded. [file 12984_2024_1360_MOESM8_ESM.docx]

**Table 1. VMR vs APM Scores after Removing Participants with Ipsilesional Proprioceptive Impairments (TLT)**

| **N = 36** | **Initial Adaptation** | **Final Adaptation** | **Trials to Adapt** |
| --- | --- | --- | --- |
| **APM Score**  **(Spearman correlation)** | rho = 0.189, (*p* = 0.269) | rho = 0.135 (*p* = 0.433) | rho = 0.066 (*p* = 0.702) |
| **APM Score**  **(Fisher’s Exact test)** | OR = 3.00, (*p* = 0.605) | OR = 1.161, (*p* = 1.00) | OR = 1.33, (*p* = 0.742) |

Note: p-values are Bonferonni-Holm corrected.

**Table 2. VMR vs Individual APM Variables after Removing Participants with Ipsilesional Proprioceptive Impairments (TLT)**

| **N = 37** | **Initial Adaptation** | **Final Adaptation** | **Trials to Adapt** | **AE XY** | **Var XY** | **Area XY** | **Shift XY** |
| --- | --- | --- | --- | --- | --- | --- | --- |
| **Initial Adapt** |  | rho = 0.127 (*p* = 0.453) | rho = -0.411 (*p* = 0.217) | rho = 0.212 (*p* = 0.207) | rho = -0.080 (*p* = 0.637) | rho = -0.115 (*p* = 0.588) | rho = 0.201 (*p* = 0.370) |
| **Final Adapt** | OR = 1.04, (*p* = 1.00) |  | rho = -0.477 (*p* = 0.054) | rho = 0.223 (*p* = 0.184) | rho = 0.158 (*p* = 0.351) | rho = 0.043 (*p* = 0.110) | rho = 0.160 (*p* = 0.0105)* |
| **Trials to Adapt** | OR = 6.00, (*p* = 0.142) | OR = 10.5, (*p* = 0.266) |  | rho = -0.021 (*p* = 0.901) | rho = 0.090 (*p* = 0.597) | rho = 0.009 (*p* = 0.958) | rho = -0.108 (*p* = 0.527) |
| **AE XY** | OR = 6.00, (*p* = 0.142) | OR = 1.44, (*p* = 0.705) | OR = 1.41, (*p* = 0.732) |  | rho = 0.661 (*p* < 0.001)* | rho = -0.309 (*p* = 0.063) | rho = 0.734 (*p* < 0.001)* |
| **Var XY** | OR = 5.25, (*p* = 0.283) | OR = 0.667, (*p* = 0.711) | OR = 1.88, (*p* = 0.493) | OR = 40.0, (*p* < 0.001)* |  | rho = -0.140 (*p* = 0.410) | rho = 0.160 (*p* 0.344) |
| **Area XY** | OR = 1.20, (*p* = 1.00) | OR = 0.923, (*p* = 1.00) | OR = 1.30, (*p* = 0.745) | OR = 2.07, (*p* = 0.328) | OR = 4.29, (*p* = 0.847) |  | rho = -0.090 (*p* = 0.597) |
| **Shift XY** | OR = 4.50, (*p* = 0.198) | OR = 0.375, (*p* = 0.649) | OR = 0.982, (*p* = 1.00) | OR = 22.0, (*p* = 0.04)* | OR = 6.67, (*p* = 0.758) | OR = 1.23, (*p* = 1.00) |  |

Note: p-values are Bonferonni-Holm corrected.

**Table 3. VMR vs AMM Scores after Removing Participants with Ipsilesional Proprioceptive Impairments (TLT)**

| **N = 35** | **Initial Adaptation** | **Final Adaptation** | **Trials to Adapt** |
| --- | --- | --- | --- |
| **AMM Score**  **(Spearman correlation)** | rho = 0.216 (*p* = 0.212) | rho = -0.078 (*p* = 0.658) | rho = 0.086 (*p* = 0.622) |
| **AMM Score**  **(Fisher’s Exact test)** | OR = 0.462 (*p* = 0.635) | OR = 4.50 (*p* = 0.112) | OR = 2.00 (*p* = 0.483) |

Note: p-values are Bonferonni-Holm corrected.

**Table 4. VMR vs Individual AMM Variables after Removing Participants with Ipsilesional Proprioceptive Impairments (TLT)**

| **N = 36** | **Initial Adaptation** | **Final Adaptation** | **Trials to Adapt** | **RL** | **SPR** | **IDE** | **PLR** |
| --- | --- | --- | --- | --- | --- | --- | --- |
| **Initial Adapt** |  | rho = 0.127 (*p* = 0.453) | rho = -0.411 (*p* = 0.217) | rho = 0.213 (*p* = 0.213) | rho = 0.150 (*p* = 0.381) | rho = 0.128 (*p* = 0.455) | rho = 0.127 (*p* = 0.460) |
| **Final Adapt** | OR = 1.04, (*p* = 1.00) |  | rho = -0.477 (*p* = 0.054) | rho = -0.091 (*p* = 0.596) | rho = -0.230 (*p* = 0.177) | rho = 0.155 (*p* = 0.366) | rho = 0.076 (*p* = 0.660)* |
| **Trials to Adapt** | OR = 6.00, (*p* = 0.142) | OR = 10.5, (*p* = 0.294) |  | rho = 0.378 (*p* = 0.390) | rho = 0.085 (*p* = 0.622) | rho = 0.088 (*p* = 0.608) | rho = -0.145 (*p* = 0.400) |
| **RL** | OR = 0.00, (*p* = 0.555) | OR = 2.20, (*p* = 0.384) | OR = 3.52, (*p* = 0.220) |  | rho = -0.069 (*p* = 0.690) | rho = 0.351 (*p* = 0.572) | rho = -0.070 (*p* = 0.684) |
| **SPR** | OR = 1.00, (*p* = 1.00) | OR = 1.75, (*p* = 0.660) | OR = 0.357, (*p* = 0.432) | OR = 1.00, (*p* = 1.00) |  | rho = -0.055 (*p* = 0.751) | rho = 0.622 (*p* = 0.001)* |
| **IDE** | OR = 0.556, (*p* = 1.00) | OR = 0.850, (*p* = 1.00) | OR = 0.972, (*p* = 1.00) | OR = 2.11, (*p* = 0.422) | OR = 0.850, (*p* = 1.00) |  | rho = 0.409 (*p* = 0.240) |
| **PLR** | OR = 0.00, (*p* = 0.274) | OR = 1.60, (*p* = 0.693) | OR = 0.578, (*p* = 0.501) | OR = 9.00, (*p* = 0.314) | OR = 2.970, (*p* = 0.235) | OR = 5.76, (*p* = 0.566) |  |

Note: p-values are Bonferonni-Holm corrected.

**Supplementary Materials 8:** Spearman’s correlations and Fisher’s exact tests examining the relationships between measures of visuomotor adaptation and *APM Task Score* (**Table 1**), visuomotor adaptation and measures derived from the APM task (**Table 2**), visuomotor adaptation and *AMM Task Score* (**Table 3**), and visuomotor adaptation and measures derived from the AMM task (**Table 4**) in a subsample of participants without ipsilesional proprioception impairments (assessed using the Thumb Localization Test).
